# Interpretable machine learning identifies immune-inflammatory and immunothrombotic biomarkers for myocardial injury and mortality risk stratification in severe pneumonia with diverse infectious etiologies

**DOI:** 10.3389/fcimb.2026.1911387

**Published:** 2026-08-26

**Authors:** Yicheng Wang, Feng Li, Tianqiang Yan, Yi Wu, Ruoxi Qi, Jiayi Zhang, Yijie Wang, Zhuowei Sun, Yu Zhang, Yang Chen

**Affiliations:** 1 Department of Biotechnology, College of Basic Medical Science, Dalian Medical University, Dalian, China; 2 Information Center, Second Affiliated Hospital of Dalian Medical University, Dalian, China; 3 Department of Mental Health and Psychology, Dalian Medical University, Dalian, China; 4 Department of Health Statistics, School of Public Health, Dalian Medical University, Dalian, China; 5 Department of Clinical Laboratory, Second Affiliated Hospital of Dalian Medical University, Dalian, China; 6 Liaoning Provincial Key Laboratory of Medical Cellular and Molecular Biology, Dalian Medical University, Dalian, China

**Keywords:** infectious etiologies, machine learning, myocardial injury, risk stratification, severe pneumonia

## Abstract

**Background:**

Severe pneumonia is frequently associated with dysregulated immune-inflammatory responses and immunothrombotic activation, contributing to myocardial injury and adverse clinical outcomes. Early identification of patients at high risk for myocardial injury and mortality across varied infectious etiologies remains challenging. We aimed to characterize inflammatory and coagulation-related signatures associated with myocardial injury and construct interpretable machine learning models for risk stratification in patients with severe pneumonia.

**Methods:**

This retrospective cohort study enrolled 287 adult patients with severe pneumonia admitted to the intensive care unit from 2018 to 2024. All patients were stratified into groups with bacterial infection, COVID-19 and bacterial co-infection, and influenza and bacterial co-infection. Clinical variables collected within 48 h after admission were analyzed using multivariable regression, competing-risk models, and interpretable machine learning. SHapley Additive exPlanations (SHAP) were used to identify key predictive features.

**Results:**

Myocardial injury was highly prevalent across all infectious subgroups. Patients with bacterial infection exhibited an exacerbated inflammatory and coagulation burden, characterized by elevated leukocyte counts, D-dimer levels, and prolonged prothrombin time. Multivariate analysis confirmed that D-dimer, prothrombin time, and creatinine were independently associated with myocardial injury. Machine learning analyses identified coagulation and inflammatory markers as major contributors to myocardial injury risk. For mortality prediction, the XGBoost model yielded optimal predictive performance with an AUC of 0.85. SHAP analysis revealed that vasopressor administration, prothrombin time, advanced cardiovascular support, age, and hypoxemia were the top prognostic determinants for mortality. Although infectious etiology was not independently associated with mortality, patients with myocardial injury and bacterial infection exhibited the poorest survival outcomes.

**Conclusion:**

Inflammatory and immunothrombotic signatures are closely associated with myocardial injury and adverse outcomes in severe pneumonia. Interpretable machine learning models exhibited promising discriminative performance for both myocardial injury and all-cause mortality in our cohort. Although the initial training dataset was derived from a single center with a relatively limited sample size, we performed temporal validation in this study, which preliminarily suggested the potential clinical applicability of these models for the early identification of high-risk patients.

## 1 Introduction

Severe pneumonia remains a critical global health threat, characterized by a high mortality rate and complex polymicrobial etiology involving bacterial, viral, and atypical pathogens (Hou et al., 2025; Khales et al., 2025; Brown et al., 2026). Patients with severe pneumonia often require intensive care unit (ICU) admission and mechanical ventilation, which leads to multisystem complications that substantially increase both treatment complexity and mortality risk (Li N. et al., 2025; Zhang Y. et al., 2025; Brown et al., 2026). Among these complications, myocardial injury is highly prevalent and holds clinical significance. Typically characterized by elevated cardiac troponin-I (cTnI) levels, myocardial injury portends a poor prognosis and correlates closely with increased mortality in patients with severe pneumonia (Katrukha and Katrukha, 2021; Naghiyev et al., 2025).

In recent years, the etiological spectrum of severe pneumonia has undergone significant changes. Besides bacterial pathogens, respiratory viruses, specifically severe acute respiratory syndrome coronavirus 2 (SARS-CoV-2) and seasonal influenza, are currently the major causes of respiratory failure and substantially elevate the risk of secondary bacterial co-infection (Jiang et al., 2025; Kami et al., 2025; Brown et al., 2026). SARS-CoV-2 was initially identified as 2019-nCoV after its emergence in Wuhan, China, where it caused severe COVID-19 pneumonia. The possible mechanisms of myocardial injury in COVID-19 could be direct damage to cardiomyocytes (Weng and Li, 2025; Zhang L. et al., 2025), systemic inflammation, myocardial interstitial fibrosis, interferon-mediated immune response, exaggerated cytokine response by type 1 and 2 helper T cells, in addition to coronary plaque destabilization and hypoxia (Li YY. et al., 2025; da Fonseca et al., 2026). Previous animal models of bacterial pneumonia have demonstrated the development of myocardial damage and cardiac injury, including cardiac scarring, via apoptosis and other cellular damage mechanisms, such as cardiomyocyte death and collagen deposition (Reyes et al., 2017). However, damage to the respiratory epithelial lining, up-regulation and exposure of receptors, dampening of immune response or enhancement of inflammation and immune dysregulation are believed to facilitate the establishment of bacterial infection in patients with viral co-infection (McCullers, 2006). Given the prognostic impact of myocardial injury on severe pneumonia outcomes, most retrospective cohort studies have focused on screening robust prognostic biomarkers and developing predictive models to stratify mortality risk among critically ill patients complicated with pneumonia-associated myocardial injury.

Previous real-world studies have identified multiple clinical and laboratory prognostic biomarkers for severe pneumonia outcomes using conventional statistical strategies, including logistic regression and Cox proportional hazards regression. These traditional regression-based models predominantly adopt routine clinical parameters and single or composite inflammatory biomarkers to screen independent risk factors for 28-day and in-hospital mortality, providing fundamental prognostic evidence for carbapenem-resistant bacterial pneumonia, influenza- and COVID-19-associated pulmonary aspergillosis (Kubiliute et al., 2024), and Omicron-related bacterial co-infection (Jiang et al., 2025; Kami et al., 2025; Zhang Y. et al., 2025). However, such conventional analytical methods are limited by low-dimensional variable inclusion, insufficient nonlinear fitting capacity, and a lack of quantitative feature contribution evaluation, which constrain their predictive accuracy and individualized prognostic performance in critically ill pneumonia populations (Chang et al., 2025; Yu and Huang, 2025).

A previous retrospective study focusing on sepsis-induced myocardial injury and associated in-hospital mortality adopted multiple robust statistical adjustment approaches, including propensity score matching, inverse probability weighting, and doubly robust estimation, and established a rigorous machine learning analytical workflow. The enrolled cohort was randomly divided into training and testing datasets at a 7:3 ratio, with Boruta and Least Absolute Shrinkage and Selection Operator (LASSO) regression performed for feature screening, followed by hyperparameter tuning of multiple candidate machine learning algorithms (Guo et al., 2025). Among all candidate algorithms, XGBoost achieved the optimal discriminatory performance for infection-triggered myocardial injury, as demonstrated by favorable AUROC values. SHapley Additive exPlanations (SHAP) further enabled model interpretability and ranked clinically relevant predictive variables, laying a solid foundation for developing web-accessible bedside prognostic calculators. Nevertheless, cutting-edge explainable artificial intelligence (XAI) frameworks for severe infectious diseases are poorly tailored to heterogeneous cohorts with etiologically diverse severe pneumonia. Few investigations have incorporated multi-dimensional biomarkers covering systemic inflammation, coagulation dysfunction, and cardiac damage to develop disease-specific predictive models for pneumonia-associated myocardial injury and all-cause mortality. In addition, currently available AI-based models seldom embed SHAP-driven feature explanations into patient-oriented, clinically actionable prognostic systems recommended by contemporary critical care consensus guidelines (Abbas et al., 2025; Lekadir et al., 2025).

In this study, we conducted a retrospective cohort study involving 287 adults diagnosed with severe pneumonia. We employed an integrated approach that combines conventional statistical methods, such as logistic regression, Cox proportional hazards models, and competing risks models, with machine learning algorithms, including Random Forest (RF), Support Vector Machine (SVM), Gradient Boosting (GB), and XGBoost (Dehghani et al., 2025; Dirks et al., 2025; Kim et al., 2025; Pavlovic et al., 2025; Zhao et al., 2025b; Zhao Q. et al., 2025). SHAP analysis was performed to improve model interpretability by quantifying the contributions of each predictive feature (Ishikawa et al., 2025; Ku et al., 2025; Suttirat et al., 2025). The primary objectives of this study were to characterize myocardial injury profiles across three subgroups of severe pneumonia: bacterial infection, COVID-19 and bacterial co-infection, and influenza and bacterial co-infection, and to identify the relevant risk factors; to evaluate the impact of infection or co-infection patterns on in-hospital mortality and clinical outcomes; and to elucidate the interplay between myocardial injury and infection patterns in prognostic determination.

## 2 Methods

### 2.1 Study design and participants

We conducted a retrospective observational cohort study involving 287 adult patients with an admission diagnosis suggesting severe pneumonia who were admitted to the Second Affiliated Hospital of Dalian Medical University from January 2018 to January 2024. Inclusion criteria were (1): age ≥ 18 years (2), admission to the ICU for acute hypoxemic respiratory failure requiring respiratory support, including either basic respiratory support (oxygen supplementation > 50% through a face mask or non-invasive ventilation) or advanced respiratory support (invasive mechanical ventilation). Patients were categorized into three mutually exclusive groups: (3A) Primary bacterial pneumonia without laboratory evidence of viral infection (bacterial infection group); (3B) Pneumonia caused by SARS-CoV-2 (confirmed by positive nasopharyngeal or throat swab samples, or bronchoalveolar lavage fluid) with secondary bacterial co-infection (COVID-19 and bacterial co-infection group); (3C) Pneumonia caused by influenza A or B (confirmed by positive throat swab or respiratory secretions) with secondary bacterial co-infection (seasonal influenza and bacterial co-infection group). Exclusion criteria were: (1) Age < 18 years; ICU admission for a respiratory viral infection other than SARS-CoV-2 and influenza A or B; (2) Acute coronary syndrome or stress-induced (Takotsubo) cardiomyopathy; (3) Severe renal insufficiency (estimated glomerular filtration rate <30 mL/min/1.73 m²); (4) Severe hypertension (systolic blood pressure >200 mmHg); (5) Persistent tachyarrhythmia (heart rate >150 beats/min); (6) Anemia (hemoglobin <90 g/L); (7) Pre-existing congestive heart failure or left ventricular ejection fraction <50%; (8) History of stroke or pulmonary embolism; (9) Suspected or confirmed rhabdomyolysis; (10) Neuromuscular diseases; (11) Human immunodeficiency virus (HIV) infection; and (12) Treatment with immunosuppressive agents.

To further evaluate model generalizability, a temporal validation cohort comprising 117 adult patients meeting identical inclusion and exclusion criteria was additionally and consecutively enrolled at the same institution between January 2025 and June 2026, approximately one year after the closure of the original cohort. As both cohorts were enrolled from the same institution, this represents a temporal rather than a fully independent validation.

The study protocol was approved by the Ethics Committee of the Second Affiliated Hospital of Dalian Medical University and was performed in accordance with the Declaration of Helsinki (Approval No.: KY2026-248-01). Given the retrospective nature of the study, written informed consent was waived by the ethics committee.

### 2.2 Data collection

Clinical data were extracted from the electronic medical record system of the Second Affiliated Hospital of Dalian Medical University, including basic patient demographics (age, gender), underlying diseases (hypertension, diabetes, cerebrovascular disease, coronary artery disease, valvulopathy, chronic obstructive pulmonary disease (COPD), malignancy), type of organ support required during ICU admission (basic respiratory support, advanced respiratory support, intubation, advanced cardiovascular support, renal replacement therapy), laboratory findings (white blood cell count, C-reactive protein, procalcitonin, cardiac troponin-I, D-dimer, fibrinogen, prothrombin time (PT), creatinine, PaO_2_, PaCO_2_), organ dysfunction (myocardial injury, pulmonary embolism, heart failure) and treatment (vasopressors, antiarrhythmic drugs, therapeutic anticoagulation, steroids, antibiotic therapy, antifungal therapy, prophylactic anticoagulation), and in-hospital outcomes (length of hospital stay, survival status). To ensure temporal consistency, only clinical and laboratory data obtained within 48 h of intensive care unit (ICU) admission were included in the analysis.

### 2.3 Definitions of outcomes

The outcome of interest was myocardial injury during ICU admission, defined as an elevated cTnI above the 99th percentile upper reference limit, in accordance with the Fourth Universal Definition of Myocardial Infarction (Thygesen et al., 2018). cTnI concentrations were measured using a chemiluminescent microparticle immunoassay (Troponin-I assay, Abbott Ireland Diagnostics Division). In patients with multiple measurements obtained within 48 h of admission, the peak value was used for analysis, consistent with clinical practice. In this study, myocardial injury was defined as a cTnI level > 0.068 μg/L, based on the Fourth Universal Definition of Myocardial Infarction. Other study outcomes included in-hospital survival status and length of hospital stay. All outcome events were recorded until death or the data cutoff date of January 31, 2024, whichever occurred first.

### 2.4 Regression analyses and machine learning modeling

Variables with missing data rates >30% were excluded from subsequent regression, competing-risk, and machine learning analyses, although they were retained in the descriptive and baseline comparison analyses.

For the myocardial injury outcome, independent predictors were first identified using univariate and multivariate logistic regression based on complete-case analysis. Multivariate models were selected using backward stepwise selection based on the Akaike Information Criterion using the stepAIC function in the R MASS package, and 95% confidence intervals were estimated using the Wald method.

For ML development to predict myocardial injury outcome, the cohort was first randomly partitioned into training (70%) and testing (30%) subsets using outcome-stratified sampling. This partitioning was independently repeated five times using different random seeds to enhance result robustness. Within each of the five partitions, missing values were imputed once using a MICE-based algorithm (iterative imputation with posterior sampling). The imputation model was exclusively fitted on the training subset and directly applied to the matched testing subset without refitting. Similarly, parameters for Z-score standardization were only estimated from the training dataset and subsequently used to normalize the testing subset. This sequence ensured that no information from the testing subset contaminated either the imputation or standardization steps, thereby preventing data leakage between the training and testing partitions. After LASSO-based feature selection was performed within each training subset, five classification models were constructed, including logistic regression, RF, GB, XGBoost, and support vector machine with radial basis function kernel. Performance metrics were averaged across the five train-test partitions to obtain the final estimates. For survival outcomes, univariate and multivariate Cox proportional hazards regression analyses were performed using complete-case data, with backward stepwise selection applied for variable selection. The proportional hazards assumption was assessed using Schoenfeld residual plots. In addition, Fine–Gray subdistribution hazard models were performed for both in-hospital death and live ICU discharge, with the alternate event treated as a competing risk.

For survival-based ML analyses, a 5-fold cross-validation framework nested with MICE (m = 5) was implemented to minimize data leakage and improve model robustness. Unlike the myocardial injury analysis, no LASSO-based feature selection was performed for survival modeling. Four survival ML algorithms, including Cox regression, RF survival, XGBoost survival, and GB survival models, were developed, and model performance estimates were pooled across all imputed datasets.

Model discrimination was evaluated using the area under the receiver operating characteristic curve (AUC), sensitivity, specificity, accuracy, F1-score, positive predictive value, and negative predictive value. The incremental predictive value of ML models over conventional regression models was quantified using Net Reclassification Improvement (NRI) and Integrated Discrimination Improvement (IDI). Clinical utility was assessed using decision curve analysis (DCA), and model interpretability was evaluated using SHAP. To clarify the potential translational pathway, SHAP-derived feature-importance outputs are intended to be summarized as an interpretable, clinician-facing risk profile. In clinical practice, this risk profile can be embedded within an electronic health record-based decision-support interface at the time of ICU admission and presented alongside each patient’s predicted probability of myocardial injury or in-hospital mortality. Such a design enables clinicians to identify variables driving elevated risk for individual patients rapidly. However, the feasibility, workflow burden, and clinical impact of such integration were not evaluated in this study. They will require dedicated prospective implementation research before these models could be adopted in routine ICU practice.

For temporal validation, the final myocardial injury and mortality prediction models were retrained using the entire original cohort (100%, n = 287) with the same feature selection, imputation, and standardization procedures described above, and their discriminative performance was subsequently assessed in the temporal validation cohort (n = 117).

### 2.5 Statistical analysis

All statistical analyses were performed using R software (version 4.4.1). In addition, machine learning analyses were implemented in Python (version 3.13) using the scikit-learn, XGBoost, scikit-survival, and SHAP libraries, including LASSO-based feature selection, ensemble learning algorithms, cross-validation procedures, survival modeling, and SHAP-based model interpretation. All statistical tests were two-sided, and a p-value < 0.05 was considered statistically significant. The Kolmogorov–Smirnov test was used to assess data distribution. Normally distributed continuous variables were expressed as mean ± standard deviation (SD) and compared using Student’s t-test or one-way analysis of variance (ANOVA), as appropriate. Non-normally distributed continuous variables were expressed as median with interquartile range (IQR) and compared using the Wilcoxon rank-sum (Mann–Whitney U) test or Kruskal–Wallis test. Categorical variables were expressed as frequencies and percentages and compared using Pearson’s χ² test or Fisher’s exact test, as appropriate.

## 3 Results

### 3.1 Baseline characteristics

**Figure 1:**
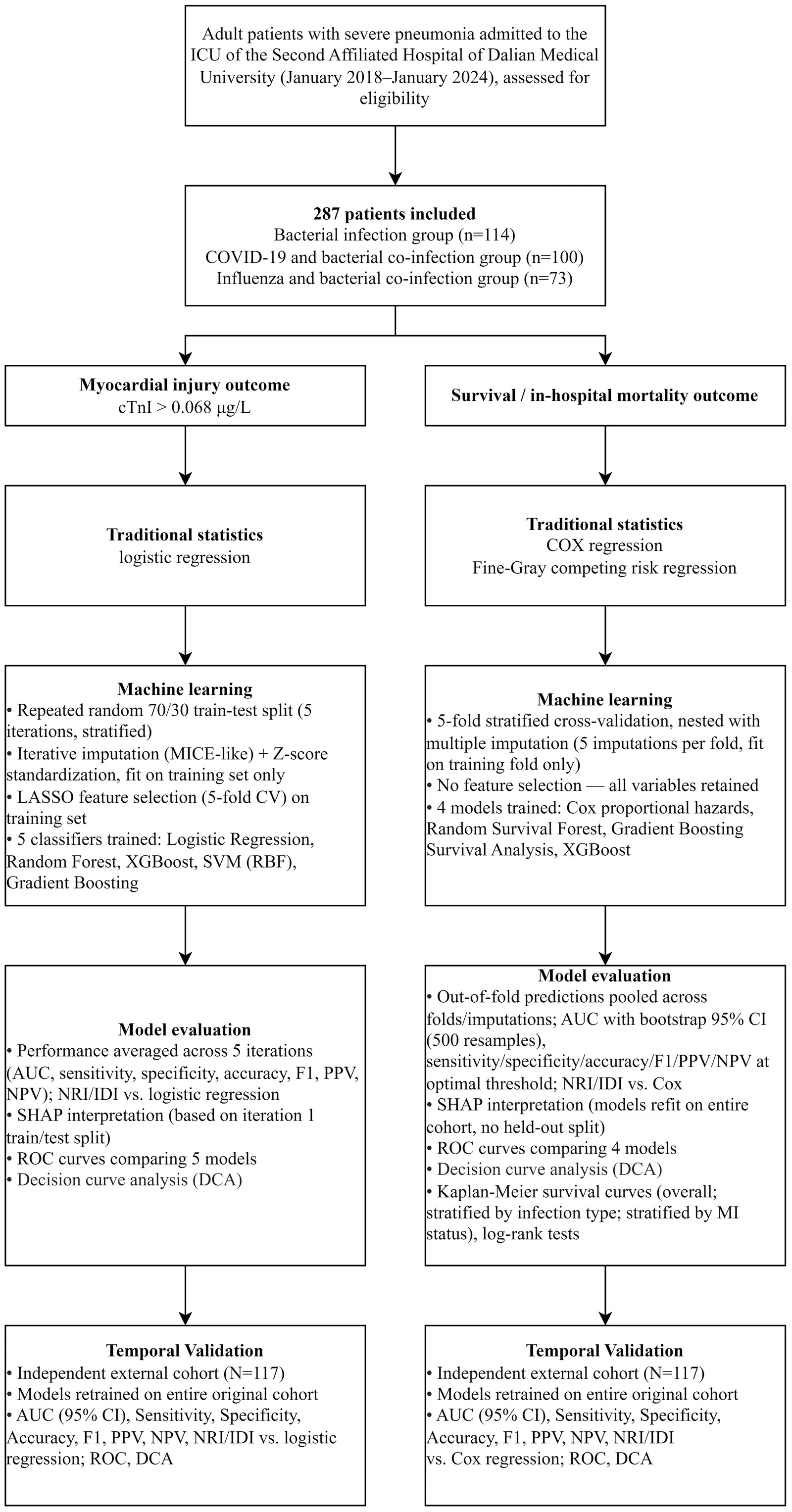
Schematic workflow illustrating patient selection, cohort grouping, model construction, internal validation, and clinical interpretation. A total of 287 enrolled patients were categorized into three infection subgroups. Two primary clinical outcomes, including myocardial injury and in-hospital mortality, were evaluated using conventional statistical methods and machine learning algorithms.

The overall workflow, including patient enrollment, model construction, internal validation, and clinical application, is illustrated in **Figure 1**. This retrospective study enrolled a total of 287 adult patients diagnosed with severe pneumonia, who were categorized into three groups: a bacterial infection group (n = 114), a COVID-19 and bacterial co-infection group (n = 100), and a seasonal influenza and bacterial co-infection group (n = 73). Baseline characteristics of the study participants are summarized in **Table 1**. Patients in the COVID-19 and bacterial co-infection group had a significantly older median age (77 [67, 85] years) compared with the bacterial infection group (75 [64, 84] years) and the seasonal influenza and bacterial co-infection group (68 [61, 79]; p = 0.035). Hypertension was significantly more prevalent in the COVID-19 and bacterial co-infection group (65.00%) than in the other two groups (p = 0.011). Patients in the bacterial infection group had higher rates of required advanced respiratory support (59.65%) and endotracheal intubation (68.42%), which were significantly higher than those in the two co-infection groups (p = 0.002 and p < 0.001, respectively). Laboratory examinations revealed significantly higher white blood cell counts (18 [12, 24] × 10^9^/L), PT (17 [15, 22] s), PaO_2_ (76 [60, 108] mmHg), and PaCO_2_ (35 ± 9 mmHg) in the bacterial infection group. Additionally, D-dimer levels were significantly higher in the bacterial infection group (4.3 [1.9, 9.1] μg/mL) and the seasonal influenza and bacterial co-infection group (4.3 [1.3, 9.0] μg/mL) than in the COVID-19 and bacterial co-infection group (p = 0.021). Overall survival time was the longest in the COVID-19 and bacterial co-infection group (15 [11, 26] days; p < 0.001) compared with that in the other two subgroups.

**Table 1:** Baseline characteristics of the study participants.

Characteristic	Overall	Bacterial infections	COVID-19 and bacterial	Seasonal influenza and bacterial	p-value
	N = 287	N = 114	N = 100	N = 73	
Age, Median (Q1, Q3)	73 (63, 84)	75 (64, 84)	77 (67, 85)	68 (61, 79)	0.035^1^
Gender, n (%)					0.848^3^
Male	201 (70.03%)	82 (71.93%)	69 (69.00%)	50 (68.49%)	
Female	86 (29.97%)	32 (28.07%)	31 (31.00%)	23 (31.51%)	
Smoking, n (%)	83 (29.75%)	33 (28.95%)	24 (24.00%)	26 (40.00%)	0.087^3^
Hypertension, n (%)	158 (56.63%)	66 (57.89%)	65 (65.00%)	27 (41.54%)	0.011^3^
Diabetes mellitus, n (%)	92 (32.97%)	41 (35.96%)	30 (30.00%)	21 (32.31%)	0.646^3^
Cerebrovascular disease, n (%)	66 (23.66%)	34 (29.82%)	18 (18.00%)	14 (21.54%)	0.114^3^
Coronary artery disease, n (%)	34 (12.19%)	18 (15.79%)	11 (11.00%)	5 (7.69%)	0.254^3^
Valvulopathy, n (%)	6 (2.15%)	2 (1.75%)	3 (3.00%)	1 (1.54%)	0.875^4^
COPD, n (%)	15 (5.38%)	10 (8.77%)	2 (2.00%)	3 (4.62%)	0.082^4^
Malignancy, n (%)	47 (16.85%)	18 (15.79%)	17 (17.00%)	12 (18.46%)	0.899^3^
White blood cell count, Median (Q1, Q3)	15 (10, 22)	18 (12, 24)	12 (8, 19)	15 (11, 22)	<0.001^1^
C-reactive protein, Median (Q1, Q3)	106 (61, 182)	105 (65, 170)	93 (49, 188)	143 (93, 185)	0.173^1^
Procalcitonin, Median (Q1, Q3)	2 (0, 9)	3 (0, 11)	2 (0, 8)	1 (0, 7)	0.104^1^
Troponin-I, Median (Q1, Q3)	0.12 (0.04, 0.42)	0.13 (0.05, 0.82)	0.09 (0.04, 0.26)	0.21 (0.04, 1.81)	0.190^1^
D-dimer, Median (Q1, Q3)	3.5 (1.3, 8.0)	4.3 (1.9, 9.1)	2.5 (1.1, 6.1)	4.3 (1.3, 9.0)	0.021^1^
Fibrinogen, Median (Q1, Q3)	5.97 (4.64, 7.39)	6.10 (4.43, 7.29)	5.89 (4.71, 7.28)	6.14 (4.87, 7.54)	0.768^1^
Prothrombin time, Median (Q1, Q3)	16 (14, 20)	17 (15, 22)	15 (14, 17)	16 (14, 20)	<0.001^1^
Creatinine, Median (Q1, Q3)	111 (69, 223)	115 (70, 243)	111 (65, 206)	97 (65, 242)	0.791^1^
PaO_2_, Median (Q1, Q3)	63 (48, 81)	76 (60, 108)	57 (44, 69)	66 (52, 78)	<0.001^1^
PaCO_2_, Mean ± SD	33 ± 8	35 ± 9	31 ± 7	34 ± 8	0.001^2^
Basic respiratory support, n (%)	162 (56.45%)	59 (51.75%)	70 (70.00%)	33 (45.21%)	0.002^3^
Advanced respiratory support, n (%)	135 (47.04%)	68 (59.65%)	39 (39.00%)	28 (38.36%)	0.002^3^
Intubation, n (%)	153 (54.84%)	78 (68.42%)	46 (46.00%)	29 (44.62%)	<0.001^3^
Vasopressors, n (%)	61 (21.86%)	42 (36.84%)	10 (10.00%)	9 (13.85%)	<0.001^3^
Advanced cardiovascular support, n (%)	34 (12.19%)	20 (17.54%)	8 (8.00%)	6 (9.23%)	0.073^3^
Renal replacement therapy, n (%)	24 (8.60%)	11 (9.65%)	10 (10.00%)	3 (4.62%)	0.423^3^
Antiarrhythmic drugs, n (%)	27 (9.68%)	19 (16.67%)	4 (4.00%)	4 (6.15%)	0.004^3^
Therapeutic anticoagulation, n (%)	89 (31.90%)	29 (25.44%)	43 (43.00%)	17 (26.15%)	0.012^3^
Steroids, n (%)	137 (49.10%)	49 (42.98%)	58 (58.00%)	30 (46.15%)	0.078^3^
Anti-bacteria, n (%)	271 (97.13%)	112 (98.25%)	95 (95.00%)	64 (98.46%)	0.419^4^
Antifungal therapy, n (%)	88 (31.54%)	41 (35.96%)	25 (25.00%)	22 (33.85%)	0.204^3^
Prophylactic anticoagulation, n (%)	11 (3.94%)	2 (1.75%)	7 (7.00%)	2 (3.08%)	0.180^4^
Overall survival time, Median (Q1, Q3)	13 (8, 21)	13 (8, 20)	15 (11, 26)	11 (8, 17)	<0.001^1^
Survival status, n (%)	74 (25.78%)	35 (30.70%)	27 (27.00%)	12 (16.44%)	0.088^3^
Myocardial injury, n (%)	90 (63.38%)	44 (65.67%)	28 (58.33%)	18 (66.67%)	0.669^3^
Pulmonary embolism, n (%)	7 (2.51%)	2 (1.75%)	3 (3.00%)	2 (3.08%)	0.793^4^
Heart failure, n (%)	11 (3.94%)	7 (6.14%)	2 (2.00%)	2 (3.08%)	0.298^4^

^1^ Kruskal-Wallis rank sum test.

^2^ One-way analysis of means.

^3^ Pearson’s Chi-squared test.

^4^ Fisher’s exact test.

### 3.2 Incidence and risk factors for myocardial injury

Univariate and multivariable logistic regression analyses were performed to identify independent risk factors for myocardial injury among patients with severe pneumonia (**Table 2**). During ICU hospitalization, myocardial injury was detected in 65.67% of patients with bacterial infection, 58.33% of those with COVID-19 and bacterial co-infection, and 66.67% of those with influenza and bacterial co-infection. Univariate analysis showed that the infection pattern was not significantly associated with the risk of myocardial injury. However, elevated D-dimer levels (OR = 1.06, 95% CI: 1.00–1.13, p = 0.044), the requirement for invasive mechanical ventilation (OR = 2.27, 95% CI: 1.13–4.58, p = 0.022), and endotracheal intubation (OR = 2.45, 95% CI: 1.18–5.08, p = 0.016) were identified as potential risk factors for myocardial injury. The relatively wide confidence intervals for these estimates reflect the limited number of outcome events, which is consistent with the overall modest effective sample size of the cohort. After adjustment for confounders, multivariable logistic regression demonstrated that D-dimer (OR = 1.12, 95% CI: 1.01–1.24, p = 0.035), PT (OR = 1.06, 95% CI: 1.00–1.13, p = 0.038), and creatinine (OR = 1.01, 95% CI: 1.00–1.01, p = 0.033) remained independent risk factors for myocardial injury. Notably, therapeutic anticoagulation was associated with an increased risk of myocardial injury (OR = 4.89, 95% CI: 1.25–19.14, p = 0.023), which may reflect the clinical management of severe coagulopathy in this patient population.

**Table 2:** Univariate and multivariable logistic regression for myocardial injury.

Characteristic	Univariate			Multivariable		
	OR	95% CI	p-value	OR	95% CI	p-value
Group						
Bacterial infections	—	—				
COVID-19 and bacterial	0.73	0.34, 1.57	0.423			
Seasonal influenza and bacterial	1.05	0.41, 2.69	0.927			
Age	1.01	0.98, 1.03	0.689			
Gender						
Male	—	—		—	—	
Female	1.36	0.64, 2.88	0.427	3.29	0.81, 13.43	0.096
Smoking (present or past)	1.71	0.75, 3.90	0.205	0.87	0.17, 4.42	0.865
Hypertension	1.54	0.76, 3.12	0.229	0.65	0.19, 2.24	0.493
Diabetes mellitus	0.79	0.39, 1.59	0.507	1.27	0.39, 4.11	0.688
Cerebrovascular disease	1.04	0.48, 2.25	0.912			
Coronary artery disease	1.28	0.51, 3.21	0.598			
Valvulopathy	3.44 ×10^6^	0.00, Inf	0.986			
COPD	4.98	0.60, 40.96	0.136			
Malignancy	0.63	0.26, 1.53	0.307			
White blood cell count (10^9^/L)	1.01	0.98, 1.05	0.396	0.97	0.92, 1.03	0.344
C-reactive protein (mg/L)	1	1.00, 1.01	0.446			
Procalcitonin (ng/mL)	1.02	1.00, 1.04	0.072			
D-dimer (μg/mL)	1.06	1.00, 1.13	0.044*	1.12	1.01, 1.24	0.035*
Fibrinogen (g/L)	1.04	0.87, 1.24	0.666	1.27	0.90, 1.81	0.178
Prothrombin time (seconds)	1.03	0.99, 1.07	0.104	1.06	1.00, 1.13	0.038*
Creatinine (μmol/L)	1	1.00, 1.00	0.119	1.01	1.00, 1.01	0.033*
PaO_2_(mmHg)	1	0.99, 1.01	0.379	1	0.99, 1.02	0.5
PaCO_2_(mmHg)	0.97	0.93, 1.03	0.316	1.01	0.94, 1.09	0.762
Basic respiratory support	0.64	0.31, 1.30	0.213	0.24	0.06, 0.99	0.049*
Advanced respiratory support	2.27	1.13, 4.58	0.022*			
Intubation	2.45	1.18, 5.08	0.016*			
Vasopressors	1.15	0.53, 2.51	0.719	0.28	0.05, 1.52	0.139
Advanced cardiovascular support	3.53	0.98, 12.76	0.054	6.05	0.63, 58.47	0.12
Renal replacement therapy	1.85	0.56, 6.05	0.312	0.05	0.00, 0.98	0.048*
Antiarrhythmic drugs	0.99	0.36, 2.69	0.983			
Therapeutic anticoagulation	2.08	0.99, 4.36	0.053	4.89	1.25, 19.14	0.023*
Steroids	1.06	0.53, 2.10	0.871			
Antifungal therapy	0.7	0.35, 1.43	0.327			
Prophylactic anticoagulation	1.76	0.18, 17.36	0.629	2.61	0.17, 39.54	0.49
Pulmonary embolism	0.57	0.08, 4.16	0.578			
Heart failure	2.11	0.42, 10.55	0.364	2.38	0.09, 61.04	0.599

*p < 0.05; **p < 0.01; ***p < 0.001.

CI, Confidence Interval; OR, Odds Ratio.

### 3.3 Prognostic factors for all-cause mortality

**Figure 2:**
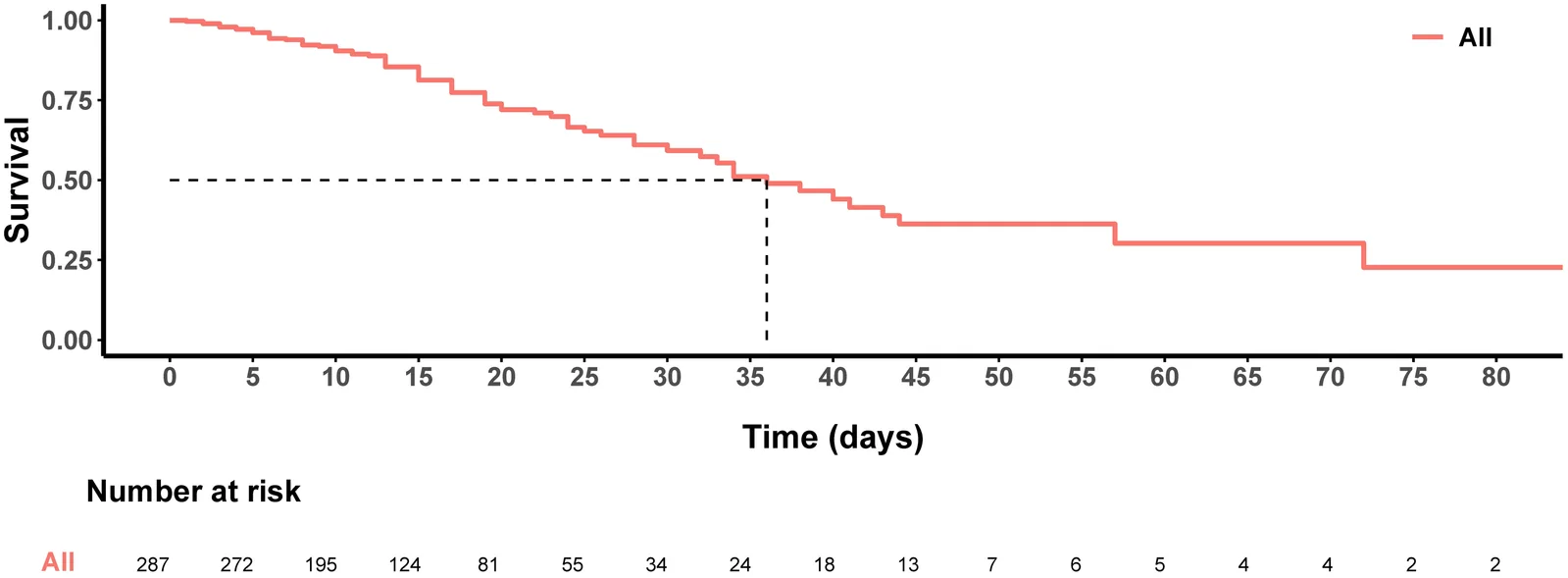
Kaplan–Meier survival curves for patients with severe pneumonia. The numbers of patients at risk at each time point are presented below the x-axis.

Kaplan–Meier (KM) survival curves showed a prominent early decline in survival within the first 30 days, followed by gradual stabilization (**Figure 2**). Univariate and multivariable Cox proportional hazards regression analyses were then performed to identify prognostic factors for all-cause mortality. After adjustment for confounding factors, endotracheal intubation (HR = 9.35, 95% CI: 3.35–26.09, p < 0.001), advanced cardiovascular support (HR = 6.78, 95% CI: 3.42–13.44, p < 0.001), fibrinogen (HR = 1.89, 95% CI: 1.59–2.26, p < 0.001), and C-reactive protein (HR = 1.01, 95% CI: 1.01–1.02, p < 0.001) were significantly associated with an increased risk of all-cause mortality. Both therapeutic and prophylactic anticoagulation emerged as independent protective factors for all-cause mortality, with HRs of 0.16 (95% CI: 0.08–0.32, p < 0.001) and 0.08 (95% CI: 0.02–0.31, p < 0.001), respectively. The relatively wide confidence intervals for these estimates likely reflect the limited number of events in each anticoagulation category. Other significant independent prognostic factors included sex and valvular heart disease. Notably, valvular heart disease was associated with a substantially elevated hazard ratio, likely reflecting the severely compromised clinical status of this patient subgroup (**Table 3**).

**Table 3:** Univariate and multivariable COX regression for survival analysis.

Characteristic	Univariate			Multivariable		
	HR	95% CI	p-value	HR	95% CI	p-value
Group						
Bacterial infections	—	—				
COVID-19 and bacterial	0.66	0.40, 1.10	0.114			
Seasonal influenza and bacterial	0.76	0.39, 1.48	0.42			
Age	1.01	0.99, 1.02	0.535			
Gender						
Male	—	—		—	—	
Female	0.84	0.50, 1.41	0.52	3.61	1.53, 8.50	0.003**
Smoking (present or past)	1.33	0.81, 2.19	0.263			
Hypertension	1.1	0.68, 1.79	0.687			
Diabetes mellitus	1.22	0.76, 1.97	0.413			
Cerebrovascular disease	1.06	0.62, 1.81	0.829	0	0.00, 0.00	<0.001***
Coronary artery disease	1.69	0.91, 3.16	0.098			
Valvulopathy	1.56	0.49, 5.00	0.453	154.33	32.05, 743.20	<0.001***
COPD	2.9	1.31, 6.38	0.008**			
Malignancy	0.79	0.41, 1.50	0.466			
White blood cell count (10^9^/L)	1.04	1.02, 1.06	<0.001***	0.95	0.93, 0.97	<0.001***
C-reactive protein (mg/L)	1	1.00, 1.00	0.222	1.01	1.01, 1.02	<0.001***
Procalcitonin (ng/mL)	1.02	1.01, 1.03	0.002**			
D-dimer (μg/mL)	1.06	1.02, 1.09	<0.001***			
Fibrinogen (g/L)	1.09	0.97, 1.22	0.158	1.89	1.59, 2.26	<0.001***
Prothrombin time (seconds)	1.03	1.01, 1.05	0.001**	1.21	1.16, 1.26	<0.001***
Creatinine (μmol/L)	1	1.00, 1.00	0.106	0.99	0.99, 0.99	<0.001***
PaO_2_ (mmHg)	1	0.99, 1.01	0.575	1.06	1.05, 1.07	<0.001***
PaCO_2 _(mmHg)	1	0.97, 1.03	0.976	1.16	1.10, 1.22	<0.001***
Basic respiratory support	0.96	0.59, 1.56	0.862			
Advanced respiratory support	1.9	1.08, 3.34	0.025*			
Intubation	1.83	0.96, 3.50	0.066	9.35	3.35, 26.09	<0.001***
Vasopressors	4.53	2.80, 7.31	<0.001***	0.06	0.03, 0.13	<0.001***
Advanced cardiovascular support	4.69	2.89, 7.60	<0.001***	6.78	3.42, 13.44	<0.001***
Renal replacement therapy	1.63	0.89, 2.99	0.116	67.17	28.29, 159.52	<0.001***
Antiarrhythmic drugs	1.28	0.69, 2.39	0.436			
Therapeutic anticoagulation	0.99	0.62, 1.60	0.979	0.16	0.08, 0.32	<0.001***
Steroids	1.64	1.00, 2.69	0.051			
Antifungal therapy	1.32	0.83, 2.11	0.245			
Prophylactic anticoagulation	0.75	0.23, 2.38	0.621	0.08	0.02, 0.31	<0.001***
Anti-bacteria	1.89	0.26, 13.64	0.528			
Pulmonary embolism	0.34	0.05, 2.48	0.287			
Heart failure	4.04	1.61, 10.16	0.003**			

*p < 0.05; **p < 0.01; ***p < 0.001.

CI, Confidence Interval; HR, Hazard Ratio.

### 3.4 Analysis of the competing risk models

In univariate Fine–Gray competing risk regression, the discharge rate was significantly higher in patients with seasonal influenza and bacterial co-infection than in those with bacterial infection (sHR = 1.58, 95% CI: 1.12–2.23; p = 0.009). However, the association was attenuated after multivariable adjustment. In contrast, COVID-19 and bacterial co-infection remained independently associated with a significantly lower hospital discharge rate (sHR = 0.45, 95% CI: 0.29–0.69; p < 0.001). Advanced cardiovascular support, increased use of vasopressors, elevated white blood cell count, and prolonged PT remained independently associated with a reduced likelihood of discharge (p < 0.05). In contrast, elevated PaCO_2_ was associated with a modestly higher discharge hazard (sHR = 1.04, 95% CI: 1.01–1.06; p = 0.001). Myocardial injury and renal replacement therapy were not associated with discharge in the adjusted model (**Table 4**).

**Table 4:** Univariate and multivariable competing risk analyses for discharge as the primary outcome.

Characteristic	Univariate			Multivariable		
	HR	95% CI	p-value	HR	95% CI	p-value
Group						
Bacterial infections	—	—				
COVID-19 and bacterial	0.99	0.73, 1.34	0.95	0.45	0.29, 0.69	<0.001***
Seasonal influenza and bacterial	1.58	1.12, 2.23	0.009**	0.83	0.51, 1.35	0.46
Myocardial Injury	0.77	0.52, 1.14	0.19			
Age	0.99	0.98, 1.00	0.039*	0.99	0.98, 1.00	0.03*
Gender						
Male	—	—				
Female	1.05	0.80, 1.39	0.73			
Troponin-I (μg/L)	0.98	0.91, 1.06	0.61			
Smoking (present or past)	0.98	0.73, 1.32	0.9			
Hypertension	0.86	0.66, 1.13	0.28			
Diabetes mellitus	0.75	0.55, 1.01	0.054			
Cerebrovascular disease	0.94	0.69, 1.28	0.68			
Coronary artery disease	0.84	0.52, 1.34	0.46			
Valvulopathy	0.52	0.16, 1.63	0.26			
COPD	0.73	0.30, 1.75	0.47			
Malignancy	0.98	0.69, 1.38	0.89			
White blood cell count (10^9^/L)	0.94	0.92, 0.96	<0.001***	0.95	0.92, 0.98	<0.001***
C-reactive protein (mg/L)	1	1.00, 1.00	0.007**			
Procalcitonin (ng/mL)	0.99	0.98, 1.00	0.092			
D-dimer (μg/mL)	0.94	0.91, 0.98	0.001**	0.97	0.93, 1.02	0.27
Fibrinogen (g/L)	0.9	0.84, 0.97	0.006**			
Prothrombin time (seconds)	0.93	0.90, 0.97	<0.001***	0.94	0.90, 0.99	0.01*
Creatinine (μmol/L)	1	1.00, 1.00	0.004**			
PaO_2_ (mmHg)	1	1.00, 1.01	0.069			
PaCO_2_ (mmHg)	1.04	1.01, 1.06	0.002**	1.04	1.01, 1.06	0.001**
Basic respiratory support	0.62	0.48, 0.81	<0.001***			
Advanced respiratory support	0.34	0.26, 0.46	<0.001***			
Intubation	0.33	0.25, 0.44	<0.001***			
Vasopressors	0.19	0.11, 0.31	<0.001***	0.31	0.13, 0.76	0.01*
Advanced cardiovascular support	0.11	0.05, 0.25	<0.001***	0.16	0.05, 0.53	0.003**
Renal replacement therapy	0.38	0.22, 0.65	<0.001***			
Antiarrhythmic drugs	0.53	0.33, 0.86	0.009**			
Therapeutic anticoagulation	0.64	0.48, 0.85	0.002**			
Steroids	0.6	0.46, 0.79	<0.001***	0.74	0.52, 1.04	0.079
Antifungal therapy	0.53	0.40, 0.71	<0.001***			
Prophylactic anticoagulation	0.85	0.46, 1.56	0.59			
Anti-bacteria	0.65	0.30, 1.41	0.27			
Pulmonary embolism	0.94	0.57, 1.56	0.81			
Heart failure	0.67	0.27, 1.67	0.39			

*p < 0.05; **p < 0.01; ***p < 0.001.

CI, Confidence Interval; HR, Hazard Ratio.

Univariate Fine–Gray competing risk analysis showed that patients with seasonal influenza and bacterial co-infection exhibited a significantly lower risk of in-hospital mortality (sHR = 0.49, 95% CI: 0.25–0.96; p = 0.038). However, this protective effect was no longer observed after multivariable adjustment (sHR = 0.85, 95% CI: 0.40–1.81; p = 0.67), suggesting that the observed difference may be explained by other clinical variables incorporated into the model. Further multivariable competing risks analysis identified advanced cardiovascular support, a higher number of vasopressors, elevated white blood cell count, and prolonged PT as independent predictors of increased in-hospital mortality (p < 0.01). Diabetes and chronic obstructive pulmonary disease were associated with in-hospital mortality in the univariate analysis but lost independent prognostic significance in the fully adjusted model (**Table 5**).

**Table 5:** Univariate and multivariable competing risk analyses for death as the primary outcome.

Characteristic	Univariate			Multivariable		
	HR	95% CI	p-value	HR	95% CI	p-value
Group						
Bacterial infections	—	—				
COVID-19 and bacterial	0.82	0.50, 1.34	0.43	1.02	0.56, 1.86	0.96
Seasonal influenza and bacterial	0.49	0.25, 0.96	0.038*	0.85	0.40, 1.81	0.67
Myocardial Injury	1.63	0.87, 3.05	0.13			
Age	1.01	1.00, 1.03	0.11	1.02	1.00, 1.04	0.11
Gender						
Male	—	—				
Female	0.86	0.52, 1.44	0.57			
Smoking (present or past)	1.17	0.71, 1.92	0.55			
Hypertension	1.24	0.77, 1.98	0.38			
Diabetes mellitus	1.64	1.03, 2.60	0.036*			
Cerebrovascular disease	1.08	0.64, 1.84	0.77			
Coronary artery disease	1.51	0.83, 2.77	0.18			
Valvulopathy	2.39	0.75, 7.60	0.14			
COPD	2.27	1.03, 5.00	0.042*			
Malignancy	0.99	0.53, 1.83	0.97			
White blood cell count (10^9^/L)	1.05	1.04, 1.06	<0.001***	1.03	1.01, 1.04	0.006**
C-reactive protein (mg/L)	1	1.00, 1.01	0.027*			
Procalcitonin (ng/mL)	1.01	1.00, 1.02	0.009**			
Troponin-I (μg/L)	1.05	0.99, 1.12	0.11			
D-dimer (μg/mL)	1.05	1.03, 1.08	<0.001***	1.01	0.97, 1.05	0.72
Fibrinogen (g/L)	1.14	1.02, 1.28	0.019*			
Prothrombin time (seconds)	1.05	1.03, 1.07	<0.001***	1.04	1.01, 1.07	0.003**
Creatinine (μmol/L)	1	1.00, 1.00	0.001**			
PaO_2_ (mmHg)	0.99	0.98, 1.01	0.39			
PaCO_2_ (mmHg)	0.95	0.92, 0.99	0.026*	0.98	0.93, 1.02	0.32
Basic respiratory support	1.57	0.97, 2.54	0.067			
Advanced respiratory support	4.42	2.55, 7.66	<0.001***			
Intubation	4.75	2.53, 8.95	<0.001***			
Vasopressors	8.35	5.22, 13.40	<0.001***	2.8	1.47, 5.32	0.002**
Advanced cardiovascular support	8.07	5.05, 12.90	<0.001***	3.73	1.84, 7.53	<0.001***
Renal replacement therapy	2.97	1.64, 5.39	<0.001***			
Antiarrhythmic drugs	2	1.12, 3.60	0.02*			
Therapeutic anticoagulation	1.57	0.99, 2.48	0.056			
Steroids	2.12	1.31, 3.45	0.002**	1.8	0.97, 3.32	0.062
Antifungal therapy	2.14	1.35, 3.39	0.001**			
Prophylactic anticoagulation	0.98	0.34, 2.83	0.97			
Anti-bacteria	2.2	0.29, 16.60	0.45			
Pulmonary embolism	0.54	0.07, 4.25	0.56			
Heart failure	2.41	0.88, 6.58	0.087			

*p < 0.05; **p < 0.01; ***p < 0.001.

CI, Confidence Interval; HR, Hazard Ratio.

### 3.5 Development and performance evaluation of machine learning models for predicting myocardial injury

**Figure 3:**
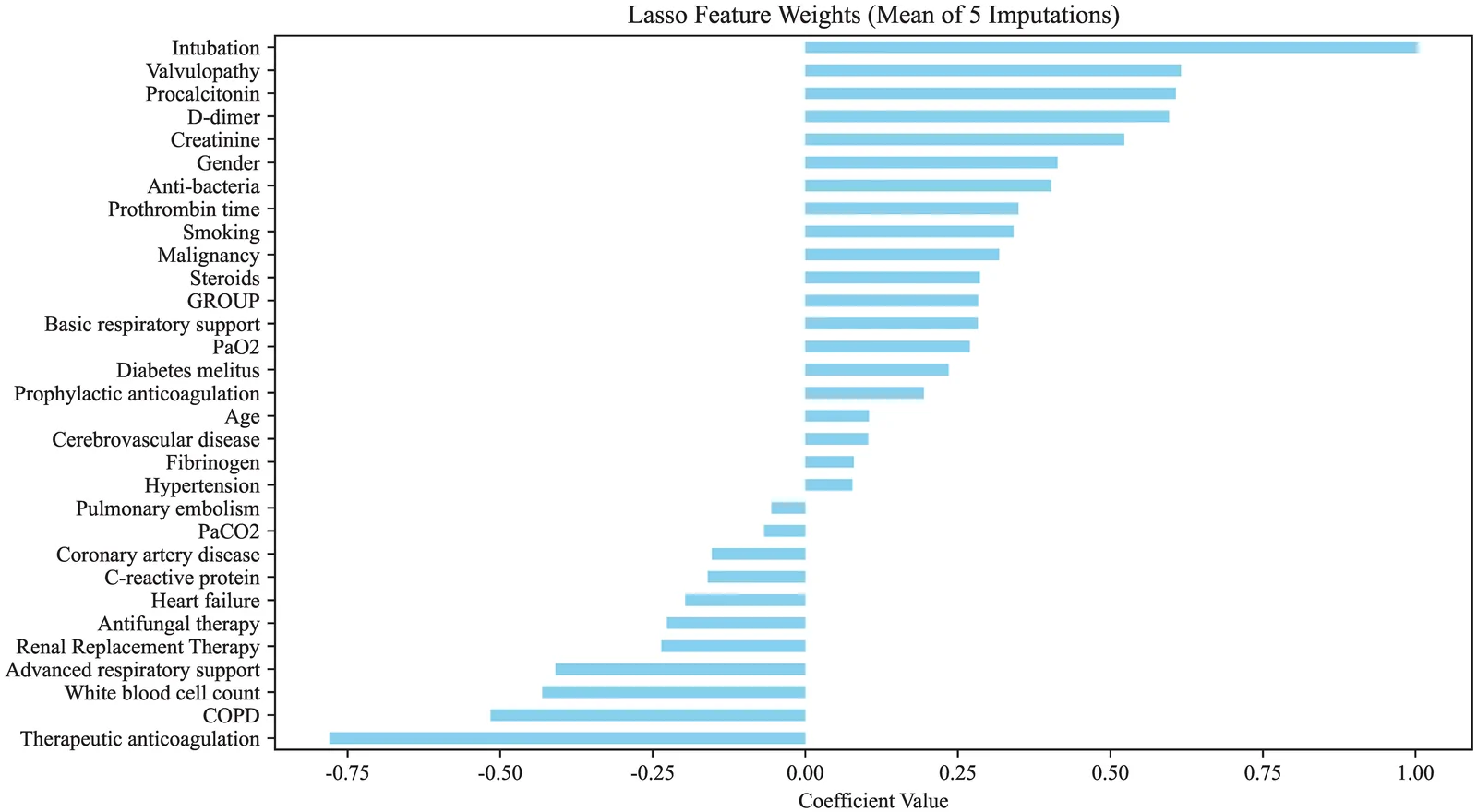
Feature selection using LASSO regression analysis for myocardial injury. LASSO regression identified key predictors of myocardial injury in severe pneumonia. Coefficients represent mean values across five imputed datasets; positive and negative values indicate risk and protective associations, respectively. Selected variables were incorporated into machine learning models.

**Figure 4:**
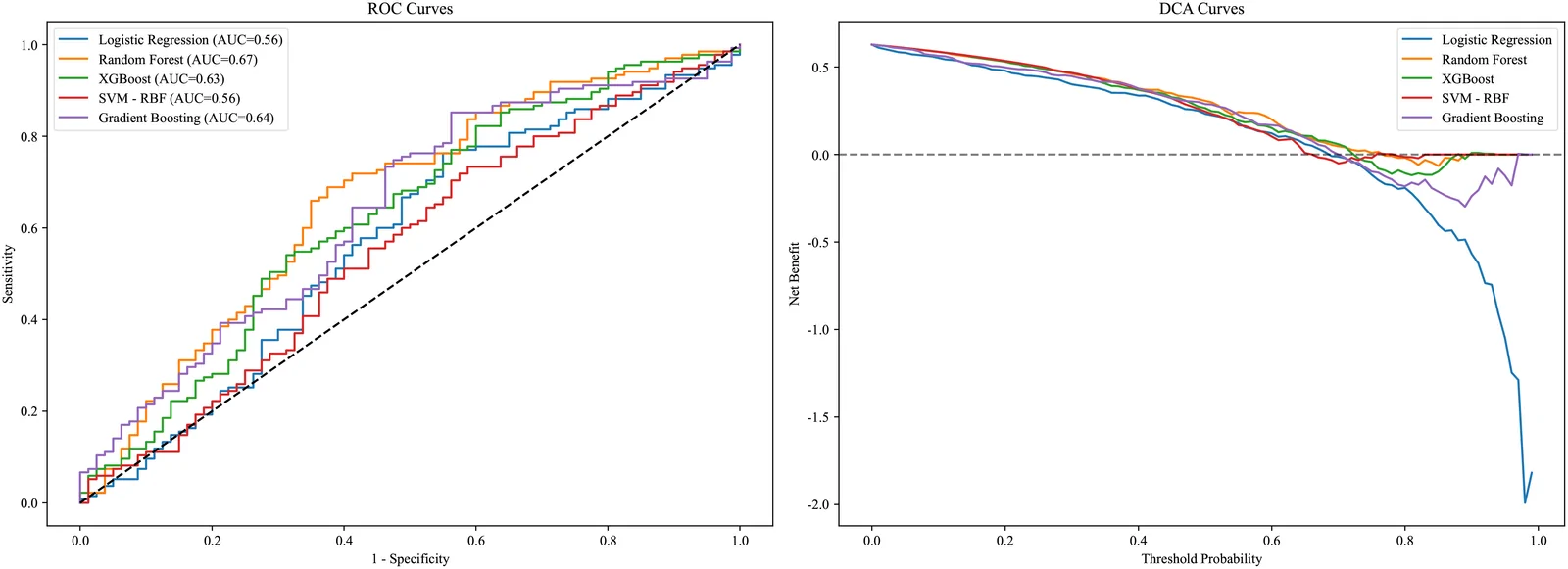
Performance and clinical utility of machine learning models for myocardial injury prediction. ROC curves (left panel) show the discriminative performance of five models, including logistic regression, random forest, XGBoost, support vector machine with RBF kernel (SVM-RBF), and gradient boosting (GB). Decision curve analysis (right panel) evaluates the net clinical benefit of each model across a range of threshold probabilities.

**Figure 5:**
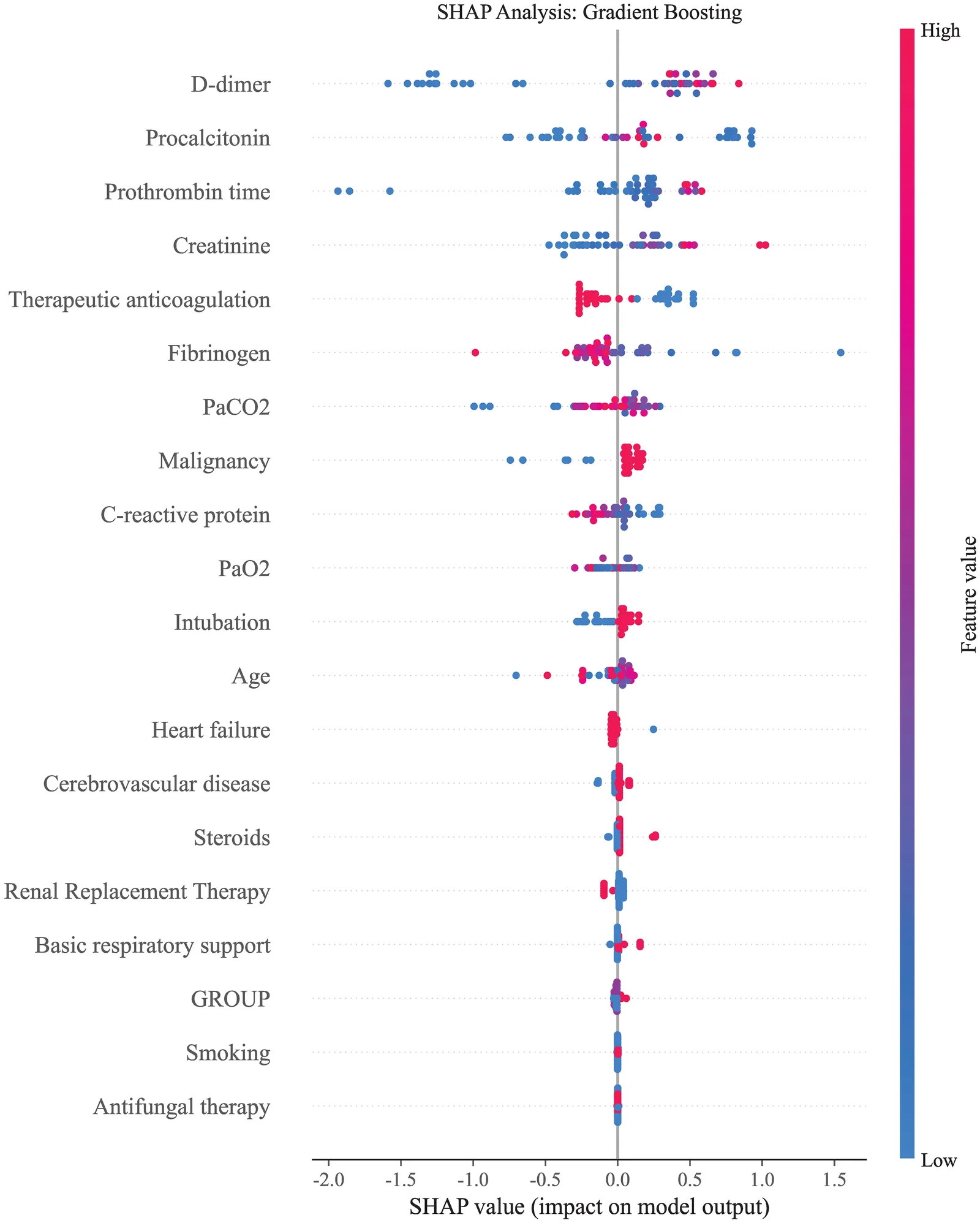
SHAP summary plot for interpretation of the gradient boosting model. SHAP analysis was used to interpret the contribution of each feature to myocardial injury prediction. Features are ranked by their overall importance. Each dot represents an individual observation, with color indicating feature value from low (blue) to high (red). The horizontal axis represents the SHAP value, indicating the direction and magnitude of each feature’s impact on the model output.

Feature selection was conducted using LASSO regression across the five imputed datasets (**Figure 3**). Therapeutic anticoagulation, intubation, D-dimer, creatinine, and PT were identified as positive predictors of myocardial injury. In contrast, basic respiratory support and renal replacement therapy (RRT) were negatively associated with myocardial injury, suggesting potential protective associations (p < 0.05). Variables with non-zero coefficients identified by LASSO were subsequently used to construct five ML models: logistic regression, RF, GB, XGBoost, and SVM. Among the 5 ML models, the RF model exhibited the best predictive performance with an AUC of 0.67 (95% CI: 0.62–0.71), followed by the GB and XGBoost models (**Figure 4**, left panel). These AUC values fell within the poor-to-moderate range according to conventional benchmarks for clinical predictive models (AUC < 0.70), indicating that, notwithstanding relative differences among the models, their absolute discriminative capacity for individual-level prediction of myocardial injury remained limited. However, the logistic regression model, SVM, and RF models exhibited lower predictive performance in the testing set. **Table 6** details the accuracy, sensitivity, specificity, negative predictive value (NPV), positive predictive value (PPV), and F1-score of each model. The GB model yielded the largest improvement in reclassification accuracy compared with the baseline logistic regression model, with an NRI of 0.12 and an IDI of 0.06. Compared with the other models, the GB model achieved a preferred balance between sensitivity (75.56%) and specificity (50.00%) and DCA (**Figure 4**, right panel) showed higher clinical utility compared with other models. Therefore, the GB model was selected as the final model architecture for subsequent clinical interpretability analysis. The SHAP summary plot for the GB model (**Figure 5**) further demonstrated the relationship between individual features and myocardial injury risk, with SHAP values greater than zero indicating a higher likelihood of myocardial injury occurrence. The top five contributing factors for high myocardial injury risk included elevated levels of D-dimer, procalcitonin, PT, creatinine, and therapeutic anticoagulation. When applied to the temporal validation cohort (n = 117), all five models retained comparable or slightly improved discrimination, with AUC values ranging from 0.64 to 0.73 (highest for RF, AUC 0.73, 95% CI: 0.63–0.83; **Supplementary Figure 1**, **Supplementary Table 2**). Given the limited size of this validation cohort, the 95% confidence intervals were correspondingly wide, indicating that validation in larger, multicenter cohorts remains warranted.

**Table 6:** Performance comparison of machine learning models for predicting myocardial injury.

Model	AUC (95% CI)	Sensitivity (%)	Specificity (%)	Accuracy (%)	F1-Score	NPV (%)	PPV (%)	NRI (vs. LR)	IDI (vs. LR)
Logistic Regression	0.56 (0.54-0.59)	67.41	48.75	60.47	0.68	47.35	68.97	-	-
Random Forest	0.67 (0.62-0.71)	85.19	38.75	67.91	0.77	62.51	70.10	-0.06	0.00
XGBoost	0.63 (0.57-0.68)	75.56	43.75	63.72	0.72	53.91	69.10	-0.11	0.01
SVM - RBF	0.56 (0.47-0.65)	88.15	25.00	64.65	0.76	58.02	66.49	-0.27	-0.07
Gradient Boosting	0.64 (0.61-0.67)	75.56	50.00	66.05	0.73	55.94	71.74	0.12	0.06

### 3.6 Development and performance evaluation of machine learning models for predicting all-cause mortality

**Figure 6:**
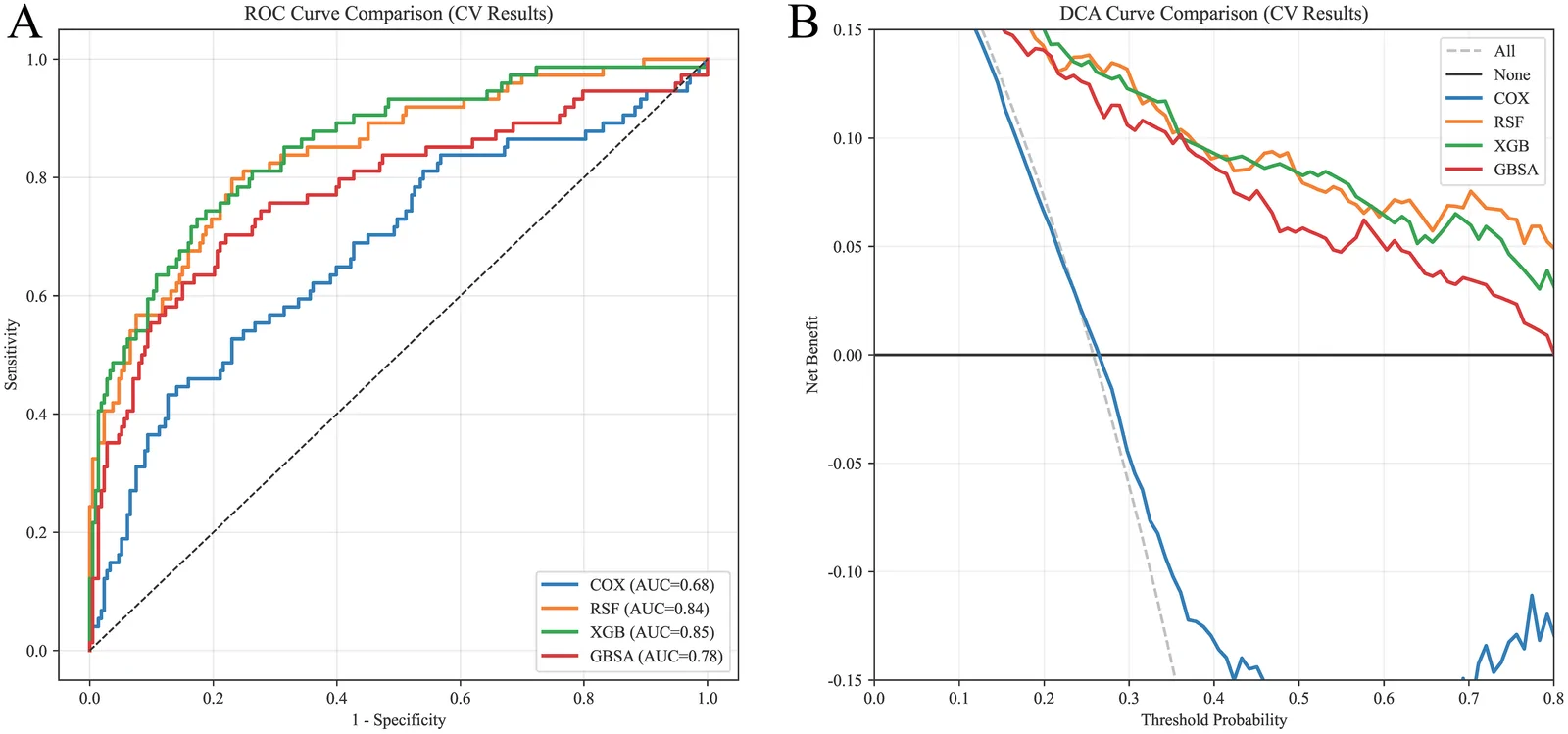
Performance comparison of survival prediction models using ROC and decision curve analysis. **(A)** Receiver operating characteristic (ROC) curves comparing the discriminative performance of Cox proportional hazards model (COX), random survival forest (RSF), XGBoost (XGB), and gradient boosting survival analysis (GBSA). XGBoost achieved the highest predictive accuracy (AUC = 0.85), followed by RSF (AUC = 0.84), GBSA (AUC = 0.78), and Cox (AUC = 0.68). **(B)** Decision curve analysis (DCA) demonstrating the clinical net benefit of each model across a range of threshold probabilities. Machine learning models, particularly XGBoost and RSF, showed superior net benefit compared to the Cox model and the default “treat-all” and “treat-none” strategies.

To evaluate the predictive performance of ML models for all-cause mortality, a 5-fold stratified cross-validation framework integrated with MICE (m = 5) was performed in the entire cohort. As illustrated in **Table 7** and **Figure 6** (left panel), the XGBoost model demonstrated the highest clinical efficiency, achieving an AUC of 0.85 (95% CI: 0.80–0.90), followed by Random Survival Forest (RSF) with an AUC of 0.84 (95% CI: 0.79–0.89) and Gradient Boosting Survival Analysis (GBSA) with an AUC of 0.78 (95% CI: 0.71–0.85). The Cox model yielded a moderate AUC of 0.68 (95% CI: 0.59–0.75), while the ensemble ML models demonstrated substantially improved predictive performance. In addition to overall accuracy, the XGBoost model showed exceptional predictive performance with a sensitivity of 72.97%, a specificity of 82.63%, and a high negative predictive value (NPV) of 89.80%. Furthermore, incremental value analysis (**Table 7**) showed that the XGBoost model significantly improved both NRI and IDI (NRI = 0.79; IDI = 0.32). DCA (**Figure 6**, right panel) further confirmed that the XGBoost model provided more substantial overall net benefits across a wide range of risk thresholds, supporting its potential to optimize risk stratification and guide therapeutic decision-making in the ICU.

**Table 7:** Predictive performance of machine learning models for survival.

Model	AUC (95% CI)	Sensitivity (%)	Specificity (%)	Accuracy (%)	F1-Score	NPV (%)	PPV (%)	NRI (vs. COX)	IDI (vs. COX)
Cox	0.68 (0.59-0.75)	43.24	87.32	75.96	0.48	81.58	54.24	-	-
Random Survival Forest	0.84 (0.79-0.89)	79.73	77.00	77.70	0.65	91.62	54.63	0.50	0.19
XGBoost	0.85 (0.80-0.90)	72.97	82.63	80.14	0.65	89.80	59.34	0.79	0.32
Gradient Boosting	0.78 (0.71-0.85)	70.27	77.93	75.96	0.60	88.30	52.53	0.28	0.14

**Figure 7:**
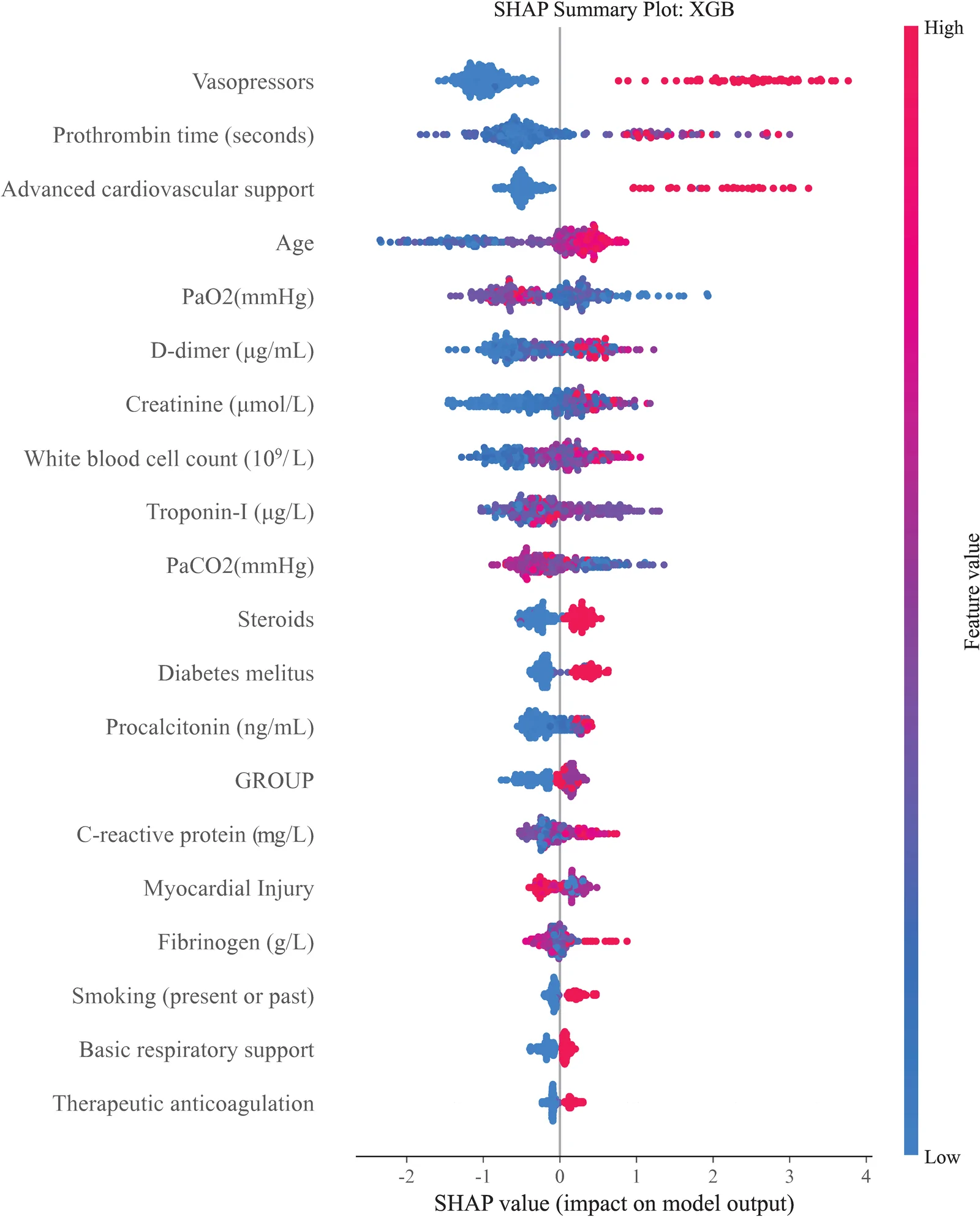
SHAP summary plot for the XGBoost model for mortality prediction. The SHAP summary plot illustrates the relative importance and association direction of all features included in the XGBoost model. Features are ranked by their mean absolute SHAP values. Each point represents an individual patient, with color indicating feature value (red: high; blue: low). Positive SHAP values correspond to increased predicted mortality risk. The number of vasopressors was the most influential predictor, followed by prothrombin time, advanced cardiovascular support, and age. Higher values of coagulation markers, inflammatory indicators, and organ dysfunction–related variables were generally associated with increased mortality risk.

The SHAP feature importance for the XGBoost model is illustrated in **Figure 7**, where the features are ranked from highest to lowest based on their mean absolute SHAP values. The five most important features were vasopressors, PT, advanced cardiovascular support, age, and PaO_2_. Laboratory findings related to organ injury including hypoxemia, elevated D-dimer, increased creatinine, white blood cell count, procalcitonin, and troponin-I were associated with higher mortality.

In the same temporal validation cohort, all four survival models likewise showed slightly improved discrimination (AUC 0.82–0.91; highest for XGBoost, AUC 0.91; **Supplementary Figure 2**, **Supplementary Table 3**), although the 95% confidence interval for the XGBoost model could not be reliably estimated owing to the limited number of events in this small sample, further underscoring the need for larger-scale, multicenter external validation.

### 3.7 Subgroup analysis and interaction testing

**Figure 8:**
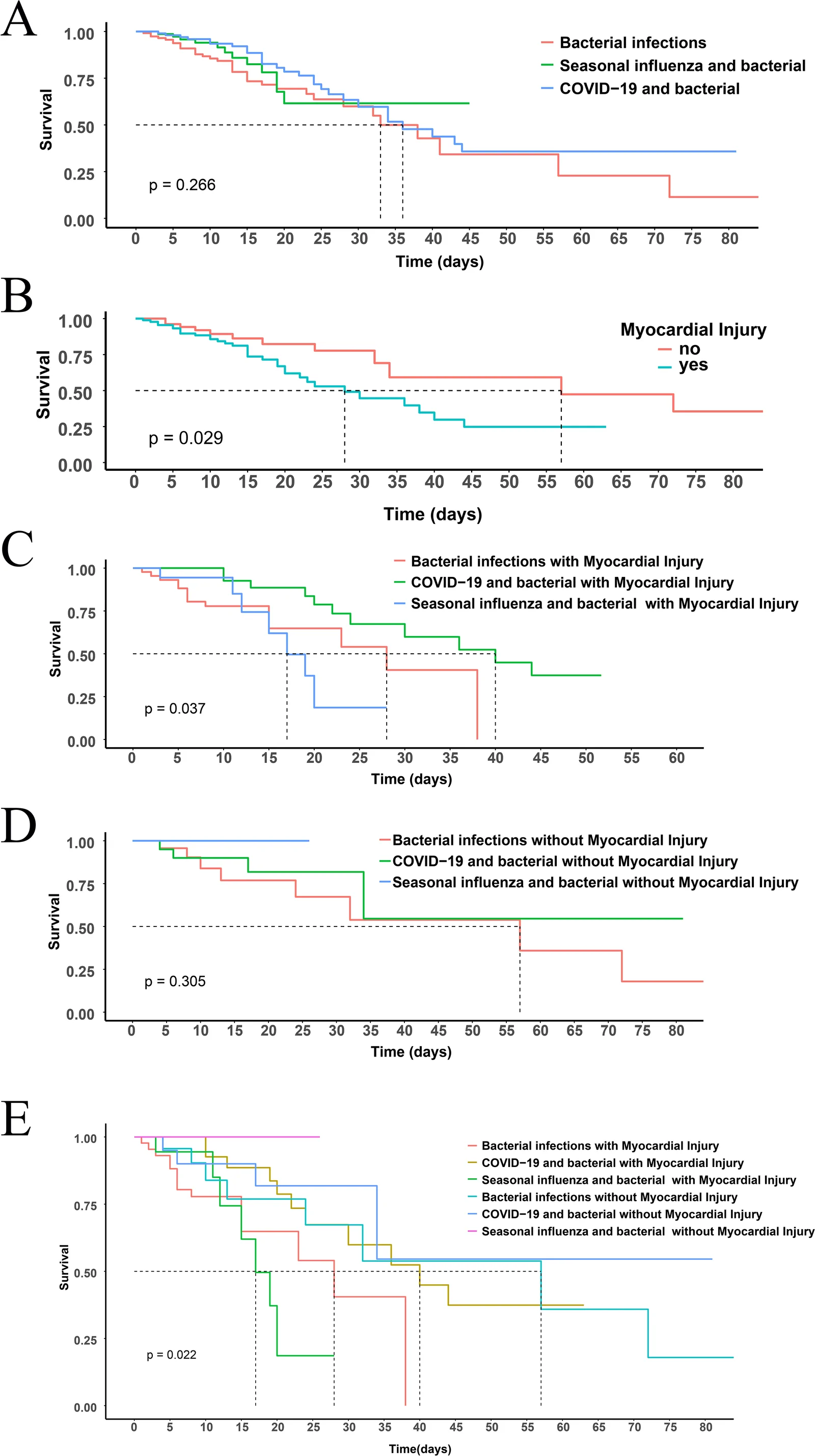
Kaplan–Meier curves of survival analyses stratified by infection type and myocardial injury status. **(A)** Overall survival stratified by infection type, showing no significant differences among groups (*p* = 0.266). **(B)** Survival stratified by the presence of myocardial injury, with significantly worse outcomes observed in patients with myocardial injury (*p* = 0.029). **(C)** Survival comparison across infection types among patients with myocardial injury, demonstrating significant differences between groups (*p* = 0.037). **(D)** Survival comparison across infection types among patients without myocardial injury, showing no significant difference (*p* = 0.305). **(E)** Combined stratification by infection type and myocardial injury status, revealing significant differences in overall survival among the six subgroups (*p* = 0.022). *P* values were calculated using the log-rank test.

The prognostic utility of the myocardial injury for predicting severe pneumonia was systematically evaluated across various patient subgroups, including myocardial injury status and infection categories. We constructed Kaplan–Meier curves to evaluate differences in survival distributions across subgroups and to explore associations between individual risk factors and survival. Survival analyses indicated that patients with myocardial injury exhibited significantly poorer survival outcomes compared to patients without myocardial injury (log-rank p = 0.029; **Figures 8A, B**). Patients in the bacterial infection group had the worst survival outcomes, while patients in the seasonal influenza and bacterial co-infection group had the most favorable survival (log-rank p = 0.037; **Figure 8C**). In contrast, there were no significant survival differences across the three infectious subgroups in patients without myocardial injury (log-rank p = 0.305; **Figure 8D**). Mortality stratified analysis revealed that the infection categories were significantly related to an increased mortality risk in patients with myocardial injury (log-rank p = 0.022; **Figure 8E**). Interaction tests showed differential associations between myocardial injury and infection categories and all-cause mortality in the patients with severe pneumonia. Specifically, the association was more significant in the patients with myocardial injury.

## 4 Discussion

The present study investigated the prognostic value of myocardial injury in patients with severe pneumonia using a combination of statistical analyses and machine learning approaches in a retrospective cohort of 287 patients. Our results demonstrate that myocardial injury represents a concomitant presentation of systemic multiple organ dysfunction syndrome and was closely correlated with adverse clinical outcomes in patients with severe pneumonia.

Baseline characteristics of 287 patients revealed distinct pathophysiological profiles between the infection categories (**Table 1**). Patients in the bacterial infection group exhibited significantly higher white blood cell counts, D-dimer levels, and PT, as well as greater requirements for invasive mechanical ventilation and vasopressor support. These laboratory findings reflect a more severe inflammatory-coagulatory burden and hemodynamic instability, which is consistent with a robust systemic inflammatory response (Ramírez et al., 2011; Goossens et al., 2022). In contrast, patients in the COVID-19 and bacterial co-infection group exhibited milder coagulation activation and a longer median survival time than those in the bacterial infection group. These findings suggest that the synergistic interplay of COVID-19 and bacterial pathogens may have triggered a distinct pattern of cardiopulmonary crosstalk, mediated by systemic inflammation, endothelial integrity, and myocardial oxygen balance. This mechanism differs substantially from the direct pathogen-driven inflammatory-coagulatory cascade observed in the bacterial infection group. Furthermore, myocardial injury was highly prevalent in the COVID-19 and bacterial co-infection group; however, no significant difference was observed among the three infection groups. This finding suggests that myocardial injury in severe pneumonia is primarily driven by systemic host responses, such as hypoxemia, cytokine storm, and microvascular dysfunction, rather than direct pathogen-mediated cardiomyocyte damage (Fajgenbaum and June, 2020; Ospina-Tascón et al., 2021; Li et al., 2022; Jacobs et al., 2023).

To further explore factors associated with myocardial injury development and its prognostic relevance in this cohort, univariable logistic regression analyses were first performed, and variables with statistical significance were subsequently entered into multivariable logistic regression models. Cox proportional hazards models were further used to assess the association between predictors and long-term outcomes. Multivariable logistic regression analysis identified that D-dimer, PT, and creatinine were independent risk factors for myocardial injury (**Table 2**). These crucial features were further screened and validated by LASSO-based feature selection (**Figure 3**). SHAP analysis of the GB model further confirmed that elevated D-dimer, procalcitonin, and PT were strongly associated with an increased risk of myocardial injury. In contrast, basic respiratory support and renal replacement therapy were correlated with a reduced myocardial injury risk (**Figure 5**). Collectively, these findings highlight the central involvement of the inflammation-coagulation cascade and renal-cardiac crosstalk in the pathophysiological mechanism underlying myocardial injury development (Johnson et al., 2019; Yang et al., 2021; Lippi et al., 2024).

Beyond the independent predictors of myocardial injury, competing-risks regression further clarified the prognostic interaction between infectious pathogens and clinical outcomes. Competing-risks analyses revealed that the crude association between infectious pathogens and in-hospital outcomes was attenuated after multivariable adjustment (**Tables 4**, **5**), indicating that the crude association between infectious pathogens and clinical outcomes is largely explained by their overlapping correlations with myocardial injury, inflammation, coagulopathy, and organ support requirements. These derangements represent manifestations reflecting overall illness severity, instead of sequential causal mediators along the causal chain. Notably, although therapeutic and prophylactic anticoagulation were independently associated with reduced mortality in multivariable Cox proportional hazards models, univariate analysis paradoxically showed an association between anticoagulation and elevated myocardial injury risk. This discrepancy is likely attributable to confounding by indication, as anticoagulants are preferentially administered to critically ill patients with severe hypercoagulability. These findings suggest that early anticoagulation may exert cardioprotective effects by preserving microvascular perfusion and mitigating thrombotic complications, rather than causing myocardial injury.

Among all machine learning algorithms constructed to predict pneumonia-related myocardial injury, the GB model yielded merely moderate discriminative performance in the internal training cohort (AUC = 0.64, 95% CI: 0.61–0.67); despite this relatively limited internal discrimination, the GB algorithm demonstrated favorable reclassification indices relative to conventional logistic regression (**Table 6**). Importantly, subsequent temporal validation achieved satisfactory predictive efficacy, greatly alleviating concerns regarding model overfitting and poor generalizability. These findings indicate that targeted methodological refinements, including algorithm fine-tuning and enrollment of larger patient cohorts can further consolidate model stability (Li et al., 2024; Pan et al., 2025). Decision curve analysis further corroborated its favorable clinical net benefit across a broad spectrum of threshold probabilities (**Figure 4**), underscoring its utility in the early identification of high-risk patients. Given that discrimination ability for myocardial injury prediction remained modest (AUC of approximately 0.64–0.67) even after temporal validation, this model should for now be regarded primarily as a hypothesis-generating tool for exploring biomarker associations rather than a ready-to-use clinical decision-support system. Regarding mortality risk stratification, ensemble machine learning models substantially outperformed the traditional Cox proportional hazards model. Notably, the XGBoost model exhibited the highest discriminative ability (AUC = 0.85), accompanied by significant improvements in both NRI and IDI (**Table 7**). Decision curve analysis confirmed that the XGBoost and random survival forest models yielded superior clinical utility compared with the Cox proportional hazards model, as well as the standard “treat-all” or “treat-none” strategies (**Figure 6**). Despite the statistically superior discriminative ability and net clinical benefit delivered by these ensemble models over the Cox regression model, it remains unclear whether such statistical improvements can be translated into tangible clinical advantages in routine ICU practice. Furthermore, ensemble machine learning algorithms require more computational resources and exhibit inherently limited interpretability compared with conventional regression frameworks. Accordingly, their incremental prognostic value relative to well-calibrated regression-derived risk scores should be carefully balanced against the elevated complexity of bedside deployment and clinical interpretation. SHAP analysis of the XGBoost model identified the number of vasopressors, PT, advanced cardiovascular support, and age as the paramount predictors of mortality (**Figure 7**). Although coagulopathy, hypoxemia, renal impairment, elevated troponin-I, and systemic inflammation were associated with an increased mortality risk, infectious pathogens played a secondary role. These findings suggest that mortality in patients with severe pneumonia is driven primarily by physiological decompensation and multi-organ dysfunction rather than by infectious etiology. Nevertheless, it is worth noting that several top-ranked predictors identified via SHAP analysis, such as the number of vasopressors administered and advanced cardiovascular supportive interventions, essentially serve as indicators of disease severity and treatment intensity, instead of early biological pathways that initiate organ injury. Their high importance in SHAP analysis therefore most likely reflects the downstream physiological burden of critical illness, rather than upstream causal pathways. This distinction must be fully taken into account when interpreting the mechanistic implications of these predictive variables.

To further explore the prognostic implications of these features, stratified survival analyses were conducted, which yielded important clinical insights (**Figure 8**). While infectious pathogens alone did not independently impact overall survival, the presence of myocardial injury acted as a significant effect modifier of prognostic outcomes. Among patients complicated with myocardial injury, those with bacterial infection exhibited significantly shorter survival than patients with viral and bacterial co-infection (log-rank p = 0.037). In contrast, no significant survival difference was observed across infectious subgroups among patients without myocardial injury (p = 0.305). These results indicate that myocardial injury acts as a critical phenotypic modifier that uncovers pathogen-specific prognostic heterogeneity. Previous studies have demonstrated that bacterial and viral co-infections typically confer poorer clinical outcomes due to synergistic pathogenic interactions. However, we observed conflicting clinical outcomes in the present cohort, which could be explained by divergent immunokinetic profiles and differences in clinical intervention regimens (Lim et al., 2019; Kim and Kim, 2022). The poor survival outcomes in patients with bacterial infection concomitant with myocardial injury may be attributable to excessive endotoxin-mediated inflammation, severe microvascular thrombosis, and marked myocardial dysfunction, which collectively fuel a vicious cycle of cardiopulmonary deterioration (Raetz and Whitfield, 2002; Reyes et al., 2017; Goossens et al., 2022). Furthermore, clinically, the intricate manifestations of co-infections often necessitate early, aggressive, and broad-spectrum empirical antimicrobial therapy. Conversely, definitive diagnosis and targeted treatment for highly virulent bacterial infections are frequently delayed due to prolonged culture turnaround times and rising antibiotic resistance, ultimately leading to irreversible myocardial injury. In contrast, despite dual pathogenicity, viral and bacterial co-infections appear to induce a less fulminant cardiac phenotype, potentially due to attenuated coagulopathy and distinct immune-inflammatory responses (Guo et al., 2020; Tschöpe et al., 2021).

SHAP analysis adopted in our study dismantles the black-box mechanism of machine learning and screened pathophysiologically validated biomarkers associated with pneumonia-related myocardial injury. SHAP quantifies the directional predictive contribution of inflammatory, coagulation, and cardiac markers to enable transparent clinical decision-making and improve model practicality. Consistent with our results, a recent study (Zhao et al., 2025a) departed from the overemphasis on predictive accuracy alone and established a multi-dimensional evaluation system incorporating algorithm fairness, model transparency, subgroup-specific feature interpretation, and bedside clinical actionability. The study constructed a complete pipeline for fair data augmentation, three-subtype classification of severe pneumonia, and stratified mortality factor identification across COVID-19 infectious, non-COVID infectious and non-infectious pneumonia, embodying the prevailing principle of robust, explainable, and patient-centered critical care AI. Notably, both investigations avoid overreliance on uninterpretable complex ensemble algorithms. Most importantly, our research delivers incremental value by extending the application of explainable AI from pneumonia subtype classification and all-cause mortality prediction to risk stratification for secondary myocardial injury. This expansion broadens the clinical scenarios of patient-centric interpretable models and provides evidence for their routine bedside translation in respiratory critical care.

In addition to advanced machine learning-based predictive paradigms illustrated above, accumulating evidence has validated routine laboratory-derived immune-inflammatory scores as simple yet powerful prognostic tools for critically ill and cardiovascular populations. Previous studies have focused on several well-recognized composite indices, including the Pan-Immune-Inflammation Value, HALP score (hemoglobin, albumin, lymphocyte, and platelet), and Naples Prognostic Score. Several analyses have confirmed that hematological and metabolic indicators can effectively predict 28-day mortality in septic patients (Uyar et al., 2026a). Furthermore, a multicenter cohort study has systematically compared the prognostic efficacy of the HALP score, Pan-Immune-Inflammation Value, and Naples Prognostic Score in critically ill septic populations (Uyar et al., 2026b). Similarly, both Pan-Immune-Inflammation Value and HALP score serve as independent mortality predictors among patients with acute coronary syndrome (Eyiol et al., 2026). These composite scoring systems synthesize conventional hematological and biochemical parameters into unified quantitative indicators, providing a cost-effective and clinically pragmatic alternative to complex machine learning-based risk stratification strategies. Therefore, integrating these validated inflammation-related indices as supplementary predictive variables, either combined with or compared against the SHAP-screened biomarkers identified in the present study, represents a promising direction for future optimization. Such improvements would further situate our findings within the growing framework of inflammation-driven prognostic modeling for critical respiratory illness.

However, several limitations of this study should be acknowledged. First, this was a single-center, retrospective observational study with a relatively limited sample size (N = 287), which may introduce selection bias and restrict the external generalizability of our findings. In addition, the modest sample size was inadequate for training complex ensemble machine learning models (such as XGBoost and RF) used for myocardial injury classification as well as survival prediction. These algorithms are inherently susceptible to overfitting when trained on small single-center datasets. Although LASSO-based feature selection, standardized data preprocessing, and 5-fold nested cross-validation and multiple imputation were applied to mitigate the overfitting risks, model performance was only assessed via temporal validation using a second cohort from the same institution (n = 117), rather than a truly independent, external multicenter cohort. The limited sample size of this validation cohort also resulted in wide 95% confidence intervals, underscoring the urgent need for further multicenter external validation. Second, myocardial injury was defined exclusively by elevated troponin-I, without confirmatory cardiac imaging or histopathological confirmation, which may underestimate or misclassify certain subtypes of myocardial injury in critically ill patients. Given that troponin elevation in severe pneumonia stems from heterogeneous pathways, including type 2 myocardial infarction and direct pathogen-induced myocardial injury, this unified myocardial injury endpoint hinders mechanistic inference of the established predictive models. Third, our predictive models incorporated only baseline admission laboratory data, thereby failing to capture the temporal fluctuations of biomarkers during ICU management. Finally, troponin, inflammatory, and coagulation markers were measured simultaneously within the 48 h window upon admission, which prevents the evaluation of their temporal causal sequence. Accordingly, myocardial injury identified in this cohort should be interpreted as a concurrent manifestation of multiple organ dysfunction syndrome, rather than a causal intermediate event. Despite these limitations, we maximized the utility of the available data using rigorous analytical methodologies. Future investigations should prioritize prospective, multicenter external validation and the construction of dynamic predictive models incorporating serially measured biomarkers to strengthen the robustness and clinical translatability of the present results. Furthermore, subsequent multicenter cohorts may explore whether the integration of explainable machine learning algorithms with well-validated inflammation-derived composite indices could further elevate predictive performance and clinical practicality.

## 5 Conclusion

In conclusion, myocardial injury is a highly prevalent and prognostically relevant complication among patients with severe pneumonia and serves as a critical indicator for risk stratification. Our findings demonstrate that routine laboratory markers, particularly cardiac troponin-I and coagulation-related parameters, can effectively identify patients at high risk for adverse clinical outcomes. Although infectious etiology alone was not an independent prognostic predictor, it substantially modified clinical outcomes in patients with myocardial injury. Specifically, bacterial infection combined with myocardial injury was independently associated with overall poorer survival.

Interpretable machine learning models integrating laboratory data delivered stronger discriminative power than conventional statistical approaches, with marked advantages in predicting all-cause mortality. These results underscore the value of laboratory-based assessments for guiding clinical management and endorse the combined use of cardiac and coagulation biomarkers to facilitate targeted monitoring and cardioprotective strategies in patients with severe pneumonia. Nevertheless, these predictive models need rigorous prospective validation in larger multicenter cohorts prior to clinical translation.

## Data Availability

The data that support the findings of this study are available on request from the corresponding author. The data are not publicly available due to privacy or ethical restrictions. Requests to access the datasets should be directed to Yang Chen, yangchen@dmu.edu.cn.
