# Supplementary material for: Interpretable machine learning identifies immune-inflammatory and immunothrombotic biomarkers for myocardial injury and mortality risk stratification in severe pneumonia with diverse infectious etiologies
Source: Front Cell Infect Microbiol. 2026 Aug 26;16:1911387. doi: 10.3389/fcimb.2026.1911387 (PMC13553397; doi:10.3389/fcimb.2026.1911387)
Supplement: Supplementary file 1 [file SupplementaryFile1.docx]

Supplementary Material

# 1 Supplementary Figures and Tables

## 1.1 Supplementary Figures

**Supplementary Figure 1.** Temporal validation of machine learning models for myocardial injury prediction.

ROC curves (A) show the discriminative performance of five models—logistic regression, random forest, XGBoost, support vector machine with RBF kernel (SVM-RBF), and gradient boosting (GB)—applied to the temporal validation cohort. Random forest achieved the highest discriminative performance (AUC = 0.73), followed by gradient boosting (AUC = 0.71) and XGBoost (AUC = 0.68), while logistic regression and SVM-RBF showed lower discrimination (AUC = 0.64 for both). Decision curve analysis (B) evaluates the net clinical benefit of each model across a range of threshold probabilities in the temporal validation cohort.

**Supplementary Figure 2.** Temporal validation of machine learning models for survival prediction.

(A) Receiver operating characteristic (ROC) curves comparing the discriminative performance of the Cox proportional hazards model (COX), random survival forest (RSF), XGBoost (XGB), and gradient boosting survival analysis (GBSA) in the temporal validation cohort. XGBoost achieved the highest predictive accuracy (AUC = 0.91), followed by RSF (AUC = 0.90), GBSA (AUC = 0.85), and Cox (AUC = 0.82). (B) Decision curve analysis (DCA) demonstrating the clinical net benefit of each model across a range of threshold probabilities in the temporal validation cohort. The ensemble machine learning models, particularly XGBoost and RSF, showed superior net benefit compared with the Cox model.

## 1.2 Supplementary Tables

**Supplementary Table 1.** Missing data summary

| Characteristic | GROUP | | | |
| --- | --- | --- | --- | --- |
|  | Overall | Bacterial infections | COVID-19 and bacterial | Seasonal influenza and bacterial |
|  | N = 287 | N = 114 | N = 100 | N = 73 |
| Age | 0 (0.0%) | 0 (0.0%) | 0 (0.0%) | 0 (0.0%) |
| Gender | 0 (0.0%) | 0 (0.0%) | 0 (0.0%) | 0 (0.0%) |
| Hypertension | 8 (2.8%) | 0 (0.0%) | 0 (0.0%) | 8 (11.0%) |
| Diabetes mellitus | 8 (2.8%) | 0 (0.0%) | 0 (0.0%) | 8 (11.0%) |
| Cerebrovascular disease | 8 (2.8%) | 0 (0.0%) | 0 (0.0%) | 8 (11.0%) |
| Advanced cardiovascular support | 8 (2.8%) | 0 (0.0%) | 0 (0.0%) | 8 (11.0%) |
| Renal replacement therapy | 8 (2.8%) | 0 (0.0%) | 0 (0.0%) | 8 (11.0%) |
| Malignancy | 8 (2.8%) | 0 (0.0%) | 0 (0.0%) | 8 (11.0%) |
| Coronary artery disease | 8 (2.8%) | 0 (0.0%) | 0 (0.0%) | 8 (11.0%) |
| Heart failure | 8 (2.8%) | 0 (0.0%) | 0 (0.0%) | 8 (11.0%) |
| Valvulopathy | 8 (2.8%) | 0 (0.0%) | 0 (0.0%) | 8 (11.0%) |
| COPD | 8 (2.8%) | 0 (0.0%) | 0 (0.0%) | 8 (11.0%) |
| Smoking (present or past) | 8 (2.8%) | 0 (0.0%) | 0 (0.0%) | 8 (11.0%) |
| D-dimer (μg/mL) | 16 (5.6%) | 4 (3.5%) | 7 (7.0%) | 5 (6.8%) |
| White blood cell count (10^9^/L) | 2 (0.7%) | 1 (0.9%) | 0 (0.0%) | 1 (1.4%) |
| Prothrombin time (seconds) | 14 (4.9%) | 3 (2.6%) | 6 (6.0%) | 5 (6.8%) |
| Creatinine (μmol/L) | 53 (18.5%) | 8 (7.0%) | 27 (27.0%) | 18 (24.7%) |
| Fibrinogen (g/L) | 16 (5.6%) | 5 (4.4%) | 6 (6.0%) | 5 (6.8%) |
| C-reactive protein (mg/L) | 77 (26.8%) | 47 (41.2%) | 6 (6.0%) | 24 (32.9%) |
| Procalcitonin (ng/mL) | 9 (3.1%) | 2 (1.8%) | 6 (6.0%) | 1 (1.4%) |
| PaO_2_ (mmHg) | 86 (30.0%) | 53 (46.5%) | 6 (6.0%) | 27 (37.0%) |
| PaCO_2_ (mmHg) | 87 (30.3%) | 53 (46.5%) | 7 (7.0%) | 27 (37.0%) |
| Basic respiratory support | 0 (0.0%) | 0 (0.0%) | 0 (0.0%) | 0 (0.0%) |
| Advanced respiratory support | 0 (0.0%) | 0 (0.0%) | 0 (0.0%) | 0 (0.0%) |
| Antiarrhythmic drugs | 8 (2.8%) | 0 (0.0%) | 0 (0.0%) | 8 (11.0%) |
| Therapeutic anticoagulation | 8 (2.8%) | 0 (0.0%) | 0 (0.0%) | 8 (11.0%) |
| Prophylactic anticoagulation | 8 (2.8%) | 0 (0.0%) | 0 (0.0%) | 8 (11.0%) |
| Steroids | 8 (2.8%) | 0 (0.0%) | 0 (0.0%) | 8 (11.0%) |
| Intubation | 8 (2.8%) | 0 (0.0%) | 0 (0.0%) | 8 (11.0%) |
| Anti-bacteria | 8 (2.8%) | 0 (0.0%) | 0 (0.0%) | 8 (11.0%) |
| Antifungal therapy | 8 (2.8%) | 0 (0.0%) | 0 (0.0%) | 8 (11.0%) |
| Vasopressors | 8 (2.8%) | 0 (0.0%) | 0 (0.0%) | 8 (11.0%) |
| Pulmonary embolism | 8 (2.8%) | 0 (0.0%) | 0 (0.0%) | 8 (11.0%) |
| Troponin-I (μg/L) | 145 (50.5%) | 47 (41.2%) | 52 (52.0%) | 46 (63.0%) |
| Myocardial injury | 145 (50.5%) | 47 (41.2%) | 52 (52.0%) | 46 (63.0%) |

**Supplementary Table 2.** Temporal validation performance of machine learning models for predicting myocardial injury

| Model | AUC (95% CI) | Sensitivity (%) | Specificity (%) | Accuracy (%) | F1-Score | NPV (%) | PPV (%) | NRI (vs. LR) | IDI (vs. LR) |
| --- | --- | --- | --- | --- | --- | --- | --- | --- | --- |
| Logistic Regression | 0.64 (0.53-0.74) | 80.65 | 36.05 | 47.86 | 0.45 | 83.78 | 31.25 | **-** | **-** |
| Random Forest | 0.73 (0.63-0.83) | 61.29 | 62.79 | 62.39 | 0.46 | 81.82 | 37.25 | -0.19 | -0.01 |
| XGBoost | 0.68 (0.57-0.79) | 48.39 | 69.77 | 64.1 | 0.42 | 78.95 | 36.59 | -0.17 | -0.01 |
| SVM - RBF | 0.64 (0.53-0.74) | 87.1 | 23.26 | 40.17 | 0.44 | 83.33 | 29.03 | -0.43 | -0.09 |
| Gradient Boosting | 0.71 (0.61-0.80) | 58.06 | 67.44 | 64.96 | 0.47 | 81.69 | 39.13 | -0.12 | 0.04 |

**Supplementary Table 3.** Temporal validation performance of machine learning models for predicting survival

| Model | AUC (95% CI) | Sensitivity (%) | Specificity (%) | Accuracy (%) | F1-Score | NPV (%) | PPV (%) | NRI (vs. COX) | IDI (vs. COX) |
| --- | --- | --- | --- | --- | --- | --- | --- | --- | --- |
| Cox | 0.82 (0.60-0.99) | 77.78 | 87.96 | 87.18 | 0.48 | 97.94 | 35 | - | - |
| Random Survival Forest | 0.90 (0.74-0.99) | 88.89 | 86.11 | 86.32 | 0.5 | 98.94 | 34.78 | -0.06 | -0.01 |
| XGBoost | 0.91 (nan-nan) | 44.44 | 97.22 | 93.16 | 0.5 | 95.45 | 57.14 | 0.19 | 0.05 |
| Gradient Boosting | 0.85 (0.70-0.97) | 66.67 | 87.04 | 85.47 | 0.41 | 96.91 | 30 | -0.06 | -0.11 |
